# The Parkinson's disease-associated genes *ATP13A2* and *SYT11* regulate autophagy via a common pathway

**DOI:** 10.1038/ncomms11803

**Published:** 2016-06-09

**Authors:** Carla F. Bento, Avraham Ashkenazi, Maria Jimenez-Sanchez, David C. Rubinsztein

**Affiliations:** 1 Department of Medical Genetics, Cambridge Institute for Medical Research (CIMR), Wellcome/MRC Building, Cambridge Biomedical Campus, Hills Road, Cambridge CB2 0XY, UK

## Abstract

Forms of Parkinson's disease (PD) are associated with lysosomal and autophagic dysfunction. ATP13A2, which is mutated in some types of early-onset Parkinsonism, has been suggested as a regulator of the autophagy–lysosome pathway. However, little is known about the ATP13A2 effectors and how they regulate this pathway. Here we show that ATP13A2 depletion negatively regulates another PD-associated gene (*SYT11*) at both transcriptional and post-translational levels. Decreased *SYT11* transcription is controlled by a mechanism dependent on MYCBP2-induced ubiquitination of TSC2, which leads to mTORC1 activation and decreased TFEB-mediated transcription of SYT11, while increased protein turnover is regulated by SYT11 ubiquitination and degradation. Both mechanisms account for a decrease in the levels of SYT11, which, in turn, induces lysosomal dysfunction and impaired degradation of autophagosomes. Thus, we propose that ATP13A2 and SYT11 form a new functional network in the regulation of the autophagy–lysosome pathway, which is likely to contribute to forms of PD-associated neurodegeneration.

## Abstract (web summary)

Mutations in ATP13A2 are associated with lysosomal dysfunction and early onset Parkinson's disease. Here Bento *et al*. show that ATP13A2 depletion negatively regulates SYT11, at both transcriptional and post-translational levels, which in turn impairs function of the autophagy-lysosome pathway.

## Graphical abstract

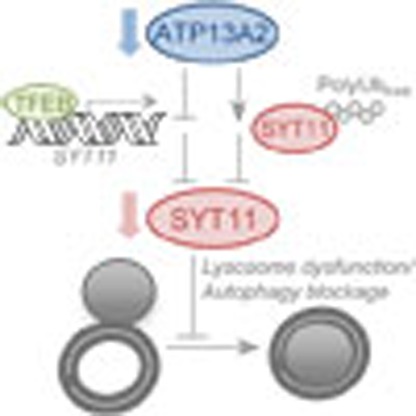

Increasing evidence has implicated autophagic dysregulation and lysosomal impairment in a wide range of neurodegenerative disorders^1^. Neurons seem to be particularly susceptible to lysosomal dysfunction, as over two-thirds of lysosomal storage diseases manifest with central nervous system dysfunction^2^. In the particular case of Parkinson's disease (PD), several disease-animal models and post-mortem brain samples of PD patients have revealed lysosomal dysfunction and accumulation of non-degraded autophagosomes in neurons, and pharmacological or genetic restoration of lysosomal degradation in such models resulted in increased autophagosome clearance and decreased dopaminergic cell death^3^,^4^,^5^.

The *ATP13A2* gene (also known as *PARK9*), which encodes a lysosomal P5-type ATPase, is mutated in autosomal recessive forms of early-onset Parkinsonism, such as the Kufor–Rabek syndrome, and thus appears to link lysosomal dysfunction and Parkinsonism^5^,^6^,^7^,^8^. Decreased levels of ATP13A2 or loss-of-function mutations that destabilize the protein in the endoplasmic reticulum (depleting it from the lysosome) impairs degradation of lysosomal substrates, diminishes lysosomal-mediated clearance of autophagosomes in dopaminergic neurons, and induces misfolding and accumulation of α-synuclein, a major protein that accumulates in neurons in PD^5^,^8^,^9^.

Several other genes, some of them still needing functional validation, have also been identified in different genome-wide association studies (GWAS) as new PD risk loci. Synaptotagmin 11 (*SYT11*) emerged as one of these putative PD-associated genes^10^,^11^,^12^,^13^ (also indicated as a candidate gene for susceptibility to schizophrenia^14^), which was also interestingly suggested as a new possible interactor of ATP13A2 in a split-ubiquitin yeast two-hybrid study^15^. Although SYT11 has been scarcely studied and reported in the literature, it belongs to a family of transmembrane proteins generally involved in vesicular fusion, trafficking and exocytosis^16^, and it was interestingly shown to localize in lysosomes in bone marrow-derived macrophages^17^, suggesting that it might regulate mechanisms relevant for autophagy and lysosome biology. Accordingly, we aimed to test whether ATP13A2 and SYT11 act in a common pathway and whether SYT11 also impacts on autophagy and/or lysosomal function, in order to provide new mechanistic insights into molecular mechanisms in PD. Here we show that ATP13A2 regulates SYT11 at both transcriptional and post-translational levels and that ATP13A2-mediated autophagy responses are dependent on SYT11, indicating that these two genes form a new functional network regulating the autophagy–lysosome pathway.

## Results

### ATP13A2 depletion decreases SYT11 mRNA expression

**Figure 1:**
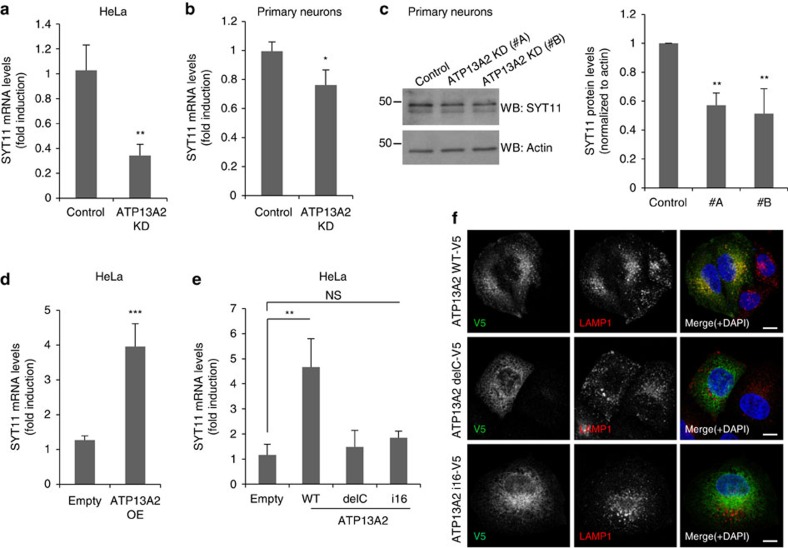
ATP13A2 regulates the mRNA expression of SYT11. (**a**) The mRNA levels of SYT11 and GAPDH in Control and ATP13A2-knockdown HeLa cells were measured by real-time PCR. The experiment (with triplicates) was repeated an additional two times and all were similarly significant. (**b**) Primary cultures of mouse neurons transduced with Control or ATP13A2 lentiviral shRNAs for 5 days were used to measure the mRNA levels of SYT11 and GAPDH by real-time PCR. The graph shows the mean±s.d. of four independent experiments. *P<0.05 (two-tailed paired Student's *t*-test). (**c**) Primary cultures of mouse neurons transduced with Control or ATP13A2 lentiviral shRNAs (#A and #B) were lysed and blotted against SYT11 and actin. A representative experiment with triplicates is shown. (**d**) The mRNA levels of SYT11 and GAPDH in HeLa cells transfected with empty pcDNA3.1 or ATP13A2 WT were measured by real-time PCR. A representative experiment (with triplicates) of three similarly significant independent experiments is shown. (**e**) HeLa cells transfected with empty pcDNA3.1, ATP13A2 WT, ATP13A2 delC or ATP13A2 i16 mutants were used to measure the mRNA levels of SYT11 and GAPDH by real-time PCR. The experiment was repeated and both experiments were similarly significant. (**f**) HeLa cells transfected with V5-tagged ATP13A2 WT, ATP13A2 delC or ATP13A2 i16 constructs were immunostained against V5 and LAMP1 and imaged using confocal microscopy (scale bar 10 μM). Unless otherwise stated, all the graphs represent mean±s.d. and statistical significance was determined using two-tailed unpaired Student's *t*-test. ^**^P<0.01; ^***^P<0.001; NS, not significant.

*SYT11* recently emerged as a new PD-associated gene, which was also suggested as a possible interactor of ATP13A2 (ref. 15). Although we were not able to detect an obvious physical interaction between SYT11 and ATP13A2 by blotting (but cannot exclude such an interaction as suggested by our mass spectrometry data) (Supplementary Fig. 1a,b), our initial results interestingly suggested that SYT11 is a downstream target of ATP13A2, since ATP13A2 knockdown decreased SYT11 mRNA levels in HeLa cells (Fig. 1a; Supplementary Fig. 1c), SH-SY5Y cells (Supplementary Fig. 1d) and primary neuronal cultures (Fig. 1b; Supplementary Fig. 1e). Although we could not examine SYT11 protein levels in human cells, as the antibodies we purchased did not work, we could confirm in mouse primary cultures of neurons that decreased SYT11 mRNA levels are accompanied by decreased SYT11 protein levels under the knockdown of ATP13A2 (Fig. 1c; Supplementary Fig. 1e). In agreement, ATP13A2 overexpression had the reverse effect, increasing the mRNA levels of SYT11 (Fig. 1d; Supplementary Fig. 1f), while overexpression of two PD-associated ATP13A2 mutants (3057 delC and 1632_1653dup22 mutants, henceforth delC and i16 mutants, respectively)^7^ were not able to increase the mRNA levels of SYT11, compared with the wild-type (WT) form (Fig. 1e; Supplementary Fig. 1f). These mutants have been described to be retained in the endoplasmic reticulum, being unable to reach the lysosome (Fig. 1f), suggesting that a fully functional ATP13A2 complex is needed at the lysosome to finely regulate SYT11 mRNA expression. (One should note that in our system, ATP13A2 loss-of-function mutants do not produce the same phenotype as ATP13A2 depletion conditions, since experiments were performed in HeLa cells, where endogenous background levels of ATP13A2 WT are sufficient to ensure normal activity of ATP13A2; this means that overexpression of loss-of-function mutants can only be compared with ATP13A2 WT overexpression conditions.) The changes observed in the regulation of *SYT11* transcription by ATP13A2 could not be attributed to apoptosis induction, as manipulation of ATP13A2 levels did not change the number of non-apoptotic and apoptotic cells as compared with control cells (Supplementary Fig. 1g).

### Regulation of SYT11 mRNA by ATP13A2 occurs via TFEB

**Figure 2:**
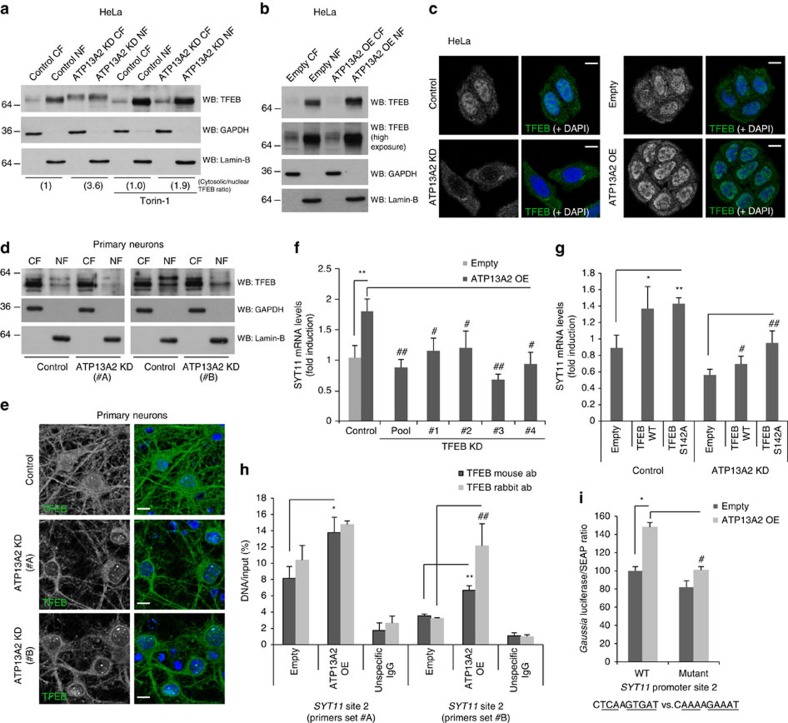
ATP13A2 controls TFEB localization and activity. (**a**,**b**) Control and ATP13A2-knockdown HeLa cells, treated with 300 nM torin-1 (or DMSO) for 4 h (**a**), and HeLa cells transfected with empty vector or ATP13A2 WT (**b**) were used for cytosolic and nuclear fractionation. The different fractions were blotted for TFEB, Lamin-B and GAPDH. (**c**) ATP13A2-knockdown cells or ATP13A2-overexpressing cells (and respective controls) growing in coverslips were fixed with cold methanol and immunostained against TFEB. TFEB translocation to the nucleus was assessed by DAPI co-localization. Cells were imaged by confocal microscopy (scale bar, 15 μM). (**d**,**e**) Primary cultures of mouse neurons were transduced with Control or ATP13A2 lentiviral shRNAs for 5 days and subsequently used for cytosolic/nuclear fractionation where the different fractions were blotted for TFEB, Lamin-B and GAPDH (**d**). Cells seeded in coverslips were also used for TFEB immunostaining (**e**) (scale bar, 10 μM). (**f**,**g**) Control and TFEB-knockdown (transfected with pool or deconvoluted oligos) HeLa cells, transfected with empty vector or ATP13A2 WT for the last 24 h (**f**), or Control and ATP13A2-knockdown HeLa cells transfected with empty pCMV, TFEB WT or TFEB S142A for the last 24 h (**g**), were used to measure the mRNA levels of SYT11 and GAPDH by real-time PCR. Representative experiments of two independent experiments with triplicates are shown. (**h**) Control and ATP13A2 WT-overexpressing HeLa cells were used to perform chromatin immunoprecipiation (ChIP). Two different antibodies were tested for TFEB-immunoprecipitation and two pairs of primers designed against the putative TFEB-binding site 2 on the promoter of *SYT11* were used for qPCR. The TFEB binding to *SYT11* promoter is represented. The experiment (with triplicates) was repeated and both experiments were similarly significant. (**i**) HeLa cells were transfected with empty pcDNA3.1 or ATP13A2 WT simultaneously with WT or CLEAR-site 2 mutant *SYT11* promoter-GLuc/SEAP. Medium was collected 48 h later. Secreted *Gaussia* luciferase and SEAP were measured. A representative experiment (with triplicates) of two independent experiments is shown. All the graphs represent mean±s.d. and statistical significance was determined using two-tailed unpaired Student's *t*-test. *P<0.05, ^**^P<0.01; ^#^*P*<0.05, ^##^*P*<0.01. CF, cytosolic fraction; NF, nuclear fraction.

To understand how SYT11 expression was regulated by ATP13A2, we analysed the DNA sequence of the human *SYT11* gene and found two putative binding sites in its promoter for the transcription factor EB (TFEB) (Supplementary Fig. 2a), a master regulator of lysosomal biogenesis^18^. The sequence of these sites fulfilled the criteria for CLEAR domains (TFEB-binding sequences) in promoter regions of genes: (i) high homology to the CLEAR consensus sequence 5′-GTCACGTGAC-3′ and (ii) the putative motifs should be located within 1,000 bp upstream and 200 bp downstream of the transcription start site^18^,^19^. As depicted in Supplementary Fig. 2a, the two plausible CLEAR sites in the *SYT11* promoter region are at −965 bp (site 1) and −543 bp (site 2) from the transcription start site, with site 2 revealing a high homology to the CLEAR consensus sequence (8 in 10 nucleotides). Interestingly, nuclear/cytosolic fractionation experiments revealed that ATP13A2 knockdown decreased the levels of TFEB in the nucleus (Fig. 2a), while ATP13A2 overexpression increased the nuclear TFEB levels (Fig. 2b), phenomena also observed by fluorescence microscopy (Fig. 2c). These changes were not due to significant changes in the total levels of TFEB (Supplementary Fig. 2b). This was further confirmed in primary mouse neuronal cultures, where ATP13A2 knockdown decreased the levels of TFEB in the nucleus (Fig. 2d,e; Supplementary Fig. 1e). Cytosolic and nuclear levels of ZKSCAN3, a master transcriptional repressor of autophagy that is thought to oppose TFEB, did not appear to be significantly changed by the manipulation of ATP13A2 levels (Supplementary Fig. 2c). Real-time PCR data allowed us to confirm that TFEB knockdown indeed decreased SYT11 mRNA levels upon ATP13A2 overexpression (Fig. 2f) (and also under basal conditions—Supplementary Fig. 2d,e), whereas overexpression of the WT or a constitutively active mutant (S142A) form of TFEB increased the SYT11 mRNA levels under ATP13A2-knockdown conditions (Fig. 2g), indicating that TFEB plays a role in the regulation of SYT11 mRNA expression by ATP13A2. Chromatin immunoprecipitation experiments also revealed that TFEB binds positively and specifically to the CLEAR domain at site 2 of *SYT11* promoter and that ATP13A2 overexpression potentiated this binding (Fig. 2h). TFEB binding to *SYT11* site 1 was not confirmed due to high unspecific binding of IgGs to chromatin at this region (Supplementary Fig. 2f). In agreement, ATP13A2 overexpression increased the transcriptional activation of the *SYT11* promoter, whereas abrogation of TFEB-binding site 2 within the *SYT11* promoter significantly reduced this effect (Fig. 2i). The fact that this effect was not fully abolished indicates that other putative TFEB-binding sites or other transcription factors might be involved in the regulation of *SYT11* transcription under these conditions. Thus, the regulation of SYT11 by ATP13A2 appears to be mediated, at least in part, by TFEB through its binding to CLEAR-site 2 in *SYT11* promoter.

**Figure 3:**
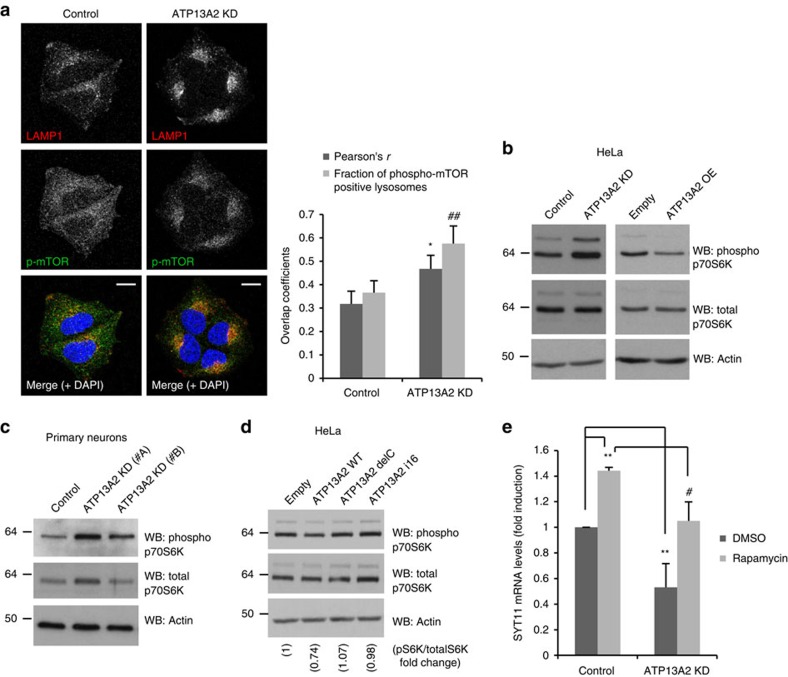
ATP13A2 regulates mTORC1 activity. (**a**) Control and ATP13A2-knockdown HeLa cells growing in coverslips were fixed and immunostained against LAMP1 and phospho-mTOR. Cells were imaged by confocal microscopy (scale bar, 15 μM). Pearson's co-localization coefficient and fraction of phospho-mTOR-positive lysosomes were determined using ImageJ. The plotted data are means±s.d. *n*=20 cells. *P<0.05; ^##^*P*<0.01 (two-tailed unpaired Student's *t*-test). The experiment was repeated an additional two times. (**b**) ATP13A2-knockdown and ATP13A2-overexpressing HeLa cells (for 96 and 24 h, respectively) were lysed and blotted for phospho-p70S6K, total-p70S6K and actin. (**c**) Primary cultures of mouse neurons were transduced with Control or ATP13A2 lentiviral shRNAs for 5 days and used for western blotting against phospho-p70S6K, total-p70S6K and actin. (**d**) HeLa cells transfected with empty vector, ATP13A2 WT, ATP13A2 delC mutant or ATP13A2 i16 mutant for 24 h were lysed and blotted against phospho-p70S6K, total-p70S6K and actin. (**e**) Control and ATP13A2-knockdown HeLa cells were treated with 100 nM rapamycin for 20 h. The mRNA levels of SYT11 and GAPDH were measured by real-time PCR. A representative experiment (with triplicates) of two independent experiments is shown. The graph represents mean±s.d. ^**^P<0.01; ^#^*P*<0.05 (two-tailed unpaired Student's *t*-test).

Serine 142 of TFEB has been characterized as a key residue for phosphorylation and regulation of TFEB by the mammalian target of rapamycin complex 1 (mTORC1), which is known to negatively regulate TFEB activity by impairing its shuttling into the nucleus^20^,^21^. Since overexpression of S142A TFEB was more effective than WT TFEB in increasing the SYT11 mRNA levels under ATP13A2-knockdown conditions (Fig. 2g), we considered that mTORC1 may be involved in the regulation of SYT11 mRNA expression by TFEB. Consistent with this model, ATP13A2 depletion induced a slight upwards shift of TFEB mobility on SDS–PAGE (polyacrylamide gel electrophoresis), compatible with increased phosphorylation, which was blunted by torin-1, a well-established mTOR inhibitor^22^ (Fig. 2a). Torin-1 treatment also tended to restore the cytosolic/nuclear TFEB ratio under the knockdown of ATP13A2 (Fig. 2a), suggesting that ATP13A2 regulates TFEB through mTORC1. In parallel, confocal microscopy data further indicated increased co-localization of phosphorylated mTOR with lysosomes/LAMP1-positive vesicles in ATP13A2-knockdown cells (Fig. 3a). This supports a model where ATP13A2 inhibition impairs TFEB activity via increased mTORC1 activity, as the latter requires localization of the mTORC1 complex at lysosomes^20^,^23^. In agreement, knockdown of ATP13A2 increased the phosphorylation of p70S6K, a well-known mTORC1 substrate frequently used as an mTORC1 activity reporter^24^, not only in HeLa cells (Fig. 3b, left panel) but also in primary neuronal cultures (Fig. 3c), while ATP13A2 overexpression decreased p70S6K phosphorylation status (Fig. 3b, right panel), indicating increased and decreased mTORC1 activity, respectively. In addition, overexpression of delC or i16 ATP13A2 mutants was ineffective in decreasing the levels of phospho-p70S6K, as compared with the WT form (Fig. 3d), suggesting that lysosomal localization of ATP13A2 is required for mTORC1 regulation. Of interest, prolonged mTORC1 inhibition by rapamycin was able to rescue the SYT11 mRNA levels under the knockdown of ATP13A2 (Fig. 3e). Thus, ATP13A2 appears to positively regulate SYT11 transcription via TFEB by inhibiting mTORC1.

### Regulation of mTORC1 by ATP13A2 occurs via TSC2

**Figure 4:**
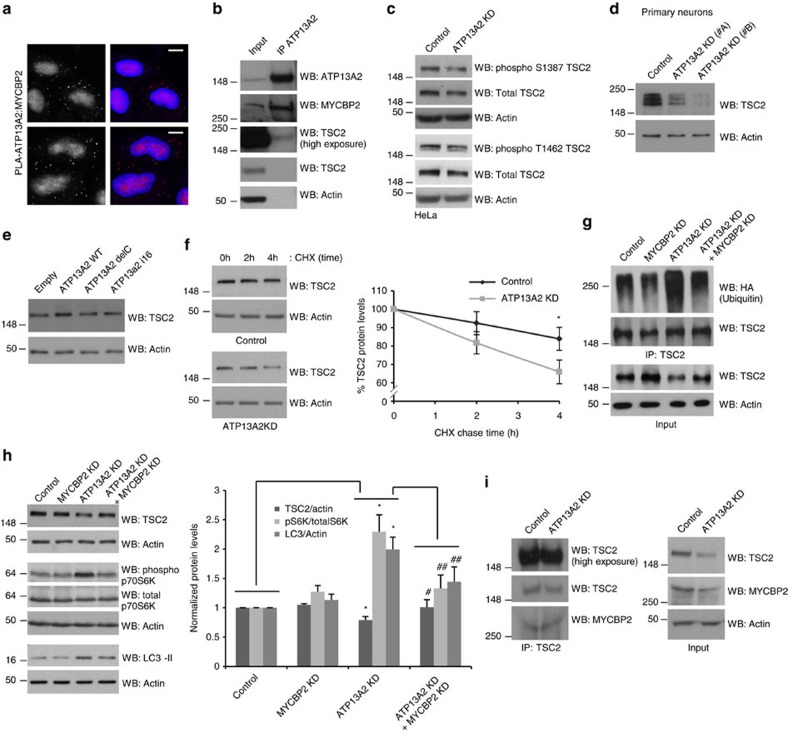
ATP13A2 regulates TSC2 through MYCBP2-induced ubiquitination. (**a**) HeLa cells were fixed and analysed by proximity ligation assay (PLA) using primary antibodies against ATP13A2 and MYCBP2. Cells were imaged by epifluorescence microscopy (scale bar, 10 μM). PLA negative controls are shown in the supplements. (**b**) Endogenous ATP13A2 was immunoprecipitated. Whole-cell lysate and immunoprecipitates were blotted against ATP13A2, MYCBP2, TSC2 and actin. (**c**) Control and ATP13A2-knockdown HeLa cells were lysed and blotted against phosphoSer1378-TSC2, phosphoThr1462-TSC2, total-TSC2 and actin. (**d**) Primary cultures of mouse neurons were transduced with Control or ATP13A2 lentiviral shRNAs for 5 days and used for western blotting against TSC2 and actin. (**e**) HeLa cells were transfected with empty vector, ATP13A2 WT, ATP13A2 delC mutant or ATP13A2 i16 mutant for 24 h. Cells were collected and cell lysates blotted for TSC2 and actin. (**f**) HeLa cells were transfected with Control or ATP13A2 siRNAs for 4 days. In the last day of transfection, cells were treated with 50 μg ml^−1^ cycloheximide for the indicated time points (after a pre-treatment of 2 h). Cell lysates were blotted against TSC2 and actin. Densitometric quantification of the bands was performed and the normalized data (TSC2 levels to actin levels) of three independent experiments is plotted in the graph, which represents mean±s.d. *P<0.05 (two-tailed paired Student's *t*-test). (**g**) HeLa cells, transfected with two rounds of either Control or ATP13A2 siRNA (50 nM) alone or in combination with MYCBP2 siRNA (50 nM) for 4 days, were further transfected with FLAG-TSC2 and HA-Ubiquitin (1:3 ratio) for 24 h. MG132 (10 μM) was added for the last 5 h. Lysates were used for TSC2 immunoprecipitation. Inputs and immunoprecipitates were blotted against HA, TSC2 and actin. (**h**) Lysates of HeLa cells transfected with Control or ATP13A2 siRNA alone or in combination with MYCBP2 siRNA (as described before) were blotted against TSC2, phospho p70S6K, total p70S6K, LC3 and actin. The graph represents the mean±s.e.m. of at least three independent experiments. *P<0.05; ^#^*P*<0.05, ^##^*P*<0.01 (two-tailed paired Student's *t*-test). (**i**) Control and ATP13A2-knockdown HeLa cells were immunoprecipitated for TSC2. Inputs and immunoprecipitates were blotted against TSC2, MYCBP2 and actin.

We hypothesized that the regulation of mTORC1 by ATP13A2 may have been due to the ATP13A2-MYCBP2 (MYC-Binding Protein 2) interaction previously reported^15^, since the latter protein is an ubiquitin ligase for Tuberous Sclerosis Complex 2 (TSC2)^25^, which in turn is a well-established negative regulator of mTORC1 (ref. 26). Indeed, a proximity ligation assay, which yields a signal when two proteins of interest are within 40 nm of each other, indicated that a fraction of MYCBP2 resides in the vicinity of ATP13A2 (Fig. 4a; Supplementary Fig. 3a). The interaction between ATP13A2 and MYCBP2 was also confirmed by endogenous ATP13A2 immunoprecipitation, where some TSC2 was also co-immunoprecipitated (Fig. 4b). A link between ATP13A2 and mTORC1 function via TSC2 was also strengthened by the observations that ATP13A2 knockdown, in HeLa cells or mouse neurons, decreased the levels of TSC2 (Fig. 4c,d), in parallel to the increased mTORC1 activity (Fig. 3b,c), and that ATP13A2 WT overexpression increased the levels of TSC2, while the delC and i16 mutants were unable to change the levels of this mTORC1 negative regulator (Fig. 4e). It should be noted that TSC2 phosphorylation followed the same trend as total TSC2 under the knockdown of ATP13A2 (Fig. 4c), suggesting that downstream signalling is likely to be affected due to lower levels of total TSC2 rather than unbalanced TSC2 phosphorylation. In agreement, depletion of ATP13A2 decreased TSC2 half-life (Fig. 4f) and induced TSC2 ubiquitination (Fig. 4g), indicating that this perturbation altered TSC2 levels by affecting protein stability. Consistent with our model where ATP13A2 inhibits MYCBP2-dependent TSC2 degradation, which decreases mTORC1 activity, MYCBP2 depletion (Supplementary Fig. 3b) re-established TSC2 ubiquitination status (Fig. 4g) and normalized the levels of TSC2 and the activity of mTORC1 under the knockdown of ATP13A2 (Fig. 4h). It should be noted that ATP13A2 depletion conditions are characterized by increased levels of LC3-II (Fig. 4h), which correlate with autophagosome numbers^27^, consistent with previous studies showing impaired lysosome function and impaired autophagosome degradation in ATP13A2-depleted cells^5^,^8^. Partial rescue of LC3-II levels by MYCBP2 knockdown in ATP13A2-depleted cells (Fig. 4h) suggests that ATP13A2 regulates the autophagy–lysosome pathway, at least in part, via TSC2 and MYCBP2. The fact that ATP13A2 depletion did not appear to obviously change the extent to which MYCBP2 interacts with TSC2 (Fig. 4i) suggests that ATP13A2 is likely to regulate MYCBP2 activity towards TSC2 rather than assisting the interaction between the two proteins.

### ATP13A2 and SYT11 depletion block autophagy

**Figure 5:**
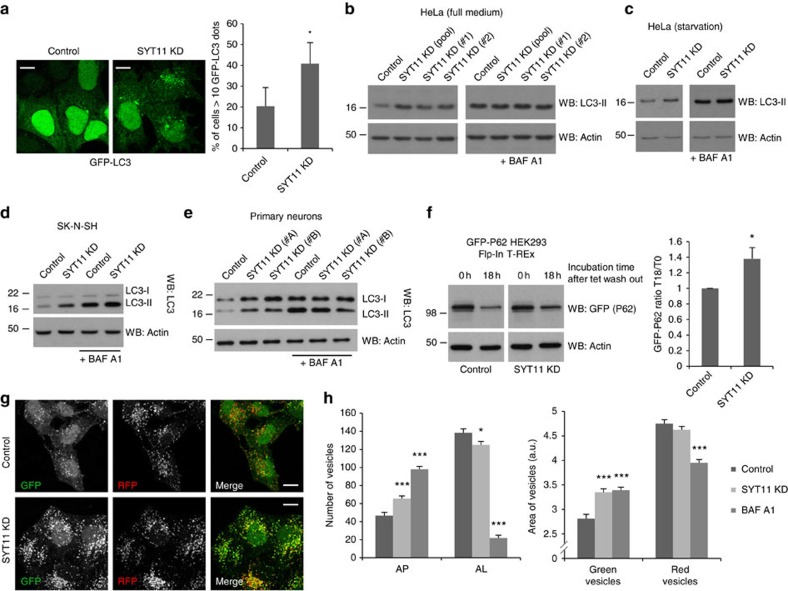
SYT11 depletion blocks autophagy. (**a**) Control and SYT11-knockdown HeLa cells were transfected with GFP-LC3 vector. Cells in coverslips were imaged by fluorescence microscopy (scale bar, 10 μM) and GFP-LC3 dots were quantified using ImageJ. The quantification shows the mean±s.d. of a minimum of 200 cells per replicate (in a total of three replicates). *P<0.05 (two-tailed unpaired Student's *t*-test). (**b**,**c**) Control and SYT11-knockdown (transfected with pool or deconvoluted oligos) HeLa cells were treated with 200 nM bafilomycin A1 (BAF A1) for the last 12 h in full medium (**b**) or with 400 nM BAF A1 for the last 4 h in HBSS medium (**c**). Cells were lysed and blotted for LC3 and actin. (**d**,**e**) Control and SYT11-knockdown SK-N-SH cells (**d**) or primary cultures of mouse neurons transduced with Control or SYT11 lentiviral shRNAs (**e**) were treated with 200 nM bafilomycin A1 (BAF A1) for the last 12 h. Lysates were blotted for LC3 and actin. (**f**) GFP-P62 HEK293 Flp-In T-REx cells were transfected with two rounds of Control or SYT11 siRNA. After the second round of transfection, GFP-P62 expression was induced by tetracycline (1 μg ml^−1^) for 24 h. Cells were then rinsed twice with PBS and incubated with normal cell culture medium (to stop transgene expression) for 18 h. Lysates were blotted for GFP and actin. The graph shows mean±s.d. of five independent experiments. *P<0.05 (two-tailed paired Student's *t*-test). (**g**,**h**) Control and SYT11-knockdown HeLa cells stably expressing tandem fluorescent-tagged LC3 (mRFP-EGFP-LC3) were fixed with 2% paraformaldehyde for 4 min and imaged by confocal microscopy (scale bar, 15 μM) (**g**) or analysed on an automated ArrayScan system (**h**). Means±s.e.m. of number of autophagosomes (AP) and autolysosomes (AL) per cell and area of green and red vesicles are shown in the graphs. BAF A1-treated cells (400 nM for 4 h) were used as a control. Approximately 2,000 cells were analysed per condition in each Cellomics experiment and the experiment was repeated three additional times. *P<0.05; ^***^P<0.001 (two-tailed paired Student's *t*-test).

Since SYT11 expression is regulated by ATP13A2, we tested whether SYT11 and ATP13A2 coordinately regulate autophagy and the lysosome. We firstly employed knockdown strategies to understand whether SYT11 regulates autophagy, by measuring the numbers of LC3 vesicles and the levels of LC3-II on SDS–PAGE gels. By overexpressing a GFP-tagged LC3 construct, we observed that SYT11 knockdown significantly increased the number of GFP-LC3 vesicles, suggesting autophagy modulation (Fig. 5a; Supplementary Fig. 4a,b). An increase of the numbers of LC3 vesicles may reflect either enhanced autophagosome synthesis, or decreased autophagosomal clearance by the lysosome. To distinguish between these scenarios, one can measure LC3-II levels under conditions where autophagosome degradation is inhibited, for example, by bafilomycin A1 (BAF A1), a potent proton pump inhibitor that blocks lysosomal degradation. Since LC3-II levels were elevated by SYT11 knockdown in the absence of BAF A1, but not in its presence (where any changes would reflect altered autophagosome biogenesis) (Fig. 5b; Supplementary Fig. 4c,d), we concluded that SYT11 knockdown blocks LC3-II/autophagosome clearance. This occurred both in full-nutrient (Fig. 5b) and starvation conditions (Fig. 5c), which stimulates autophagosome biogenesis. (Note that the amount of LC3-I detected by the antibody we used in HeLa cells is very low and can only be seen using exposures that cause oversaturation of LC3-II bands. This is not an issue, as the convention is to relate LC3-II to a loading control like actin, and not LC3-I (ref. 24). We also present the data with and without BAF A1 using different exposures, and thus distinct panels, to allow band exposures in the linear range, which is often impossible if one shows the same exposure of the samples with and without BAF A1 (refer to the full blot image in Supplementary Fig. 4d)). This result was further confirmed by the knockdown of SYT11 in SK-N-SH human neuroblastoma cells (Fig. 5d) and primary cultures of mouse neurons (Fig. 5e; Supplementary Fig. 4e). Consistent with these data, the clearance rate of the autophagic substrate P62 was decreased by the knockdown of SYT11 after switching off transgene expression in an inducible GFP-P62 HEK293 cell line (Fig. 5f).

Autophagic flux can also be assessed using an mRFP-EGFP-LC3 stable cell line, which exploits the properties of GFP and RFP; GFP fluorescence is quenched by the low pH of the lysosome when autophagosomes fuse with lysosomes, while RFP fluorescence is more stable in acidic compartments, which means that autophagosomes are labelled yellow (green+red) and autolysosomes are red only^28^. Using this cell line, we confirmed that SYT11 knockdown increased the proportion of yellow vesicles (autophagosomes), without a concomitant increase of the numbers of red-only vesicles (autolysosomes), suggesting blockage of the maturation of autophagosomes into autolysosomes (Fig. 5g,h). In agreement, automatic counting and analysis also showed a significant increase of the size of the green vesicles (Fig. 5h).

**Figure 6:**
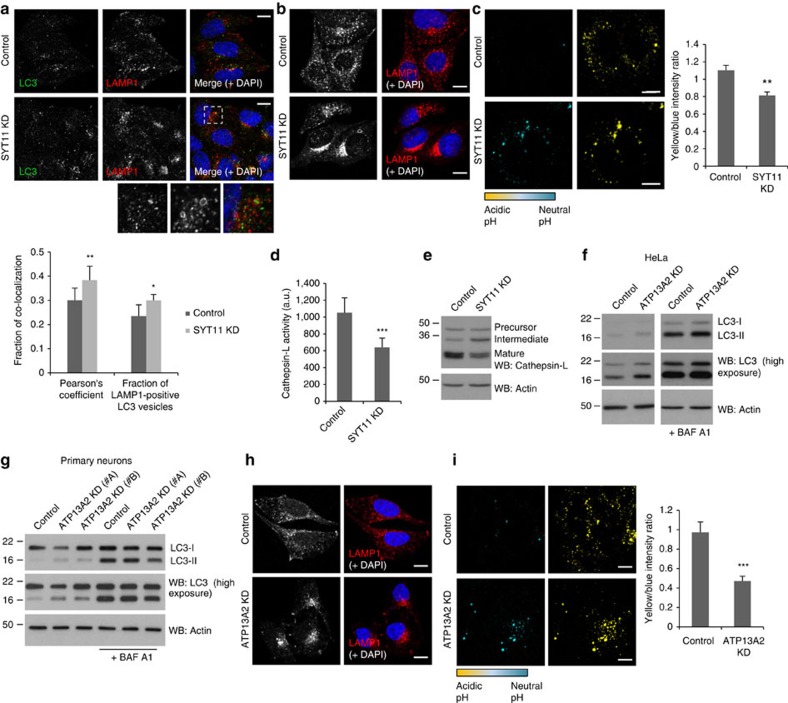
SYT11 knockdown impairs lysosomal activity similarly to ATP13A2. (**a**,**b**) Control and SYT11-knockdown HeLa cells growing in coverslips were immunostained for LAMP1 and LC3 (**a**) or only LAMP1 (**b**) and imaged by confocal microscopy (scale bar, 15 μM (**a**) or 10 μM (**b**)). Pearson's co-localization coefficient and the fraction of LAMP1-positive LC3 vesicles were determined using ImageJ. The plotted data are means±s.d. of at least 20 cells (**a**). The experiments were repeated an additional two times. (**c**) Control and SYT11-knockdown HeLa cells were loaded with LysoSensor yellow/blue and analysed by live imaging (scale bar, 5 μM). The graph shows the mean±s.e.m. of the yellow/blue intensity ratio of images obtained from 10 fields. A representative experiment of two independent experiments is shown. (**d**) Control and SYT11-knockdown HeLa cells were used to measure cathepsin-L activity *in vitro* by incubating cell lysates with 200 μM Ac-FR-AFC for 2 h at 37 °C. The graph represents fluorescence means±s.d. of three independent experiments with triplicates each condition. ^***^P<0.001 (two-tailed paired Student's *t*-test). (**e**) Lysates of control and SYT11-knockdown HeLa cells were blotted against Cathepsin-L and actin. The Cathepsin-L antibody detects the pro-form, the intermediate and the mature/processed forms of the protein. (**f**,**g**) Control and ATP13A2-knockdown HeLa cells (**f**) or primary cultures of mouse neurons transduced with Control or ATP13A2 lentiviral shRNAs (**g**) were treated with 200 nM BAF A1 for the last 12 h. Cells were lysed and blotted against LC3 and actin. (**h**) Fixed control and ATP13A2-knockdown HeLa cells were immunostained for LAMP1 and imaged by confocal microscopy (scale bar, 15 μM). (**i**) Control and ATP13A2-knockdown HeLa cells were loaded with LysoSensor yellow/blue and analysed by live imaging (scale bar, 5 μM). The graph shows the mean±s.e.m. of the yellow/blue intensity ratio of images obtained from 10 fields. A representative experiment of two independent experiments is shown. Unless otherwise stated, all the graphs represent mean±s.d. and statistical significance was determined using two-tailed unpaired Student's *t*-test. *P<0.05; ^**^P<0.01; ^***^P<0.001.

A blockage in LC3-II degradation can occur at any step after autophagosome formation and can be induced by factors such as delayed trafficking of the autophagosomes to the lysosomes, reduced fusion between both compartments and/or impaired lysosomal proteolytic activity. Impairment of autophagosome recruitment to the lysosomes can be assessed by determining LC3 co-localization with the lysosome/late endosome marker LAMP1. Confocal analysis showed that SYT11 knockdown did not decrease the co-localization of both markers, suggesting that coalescence of both compartments is not affected (Fig. 6a). SYT11 knockdown appeared indeed to increase the number of large LAMP1 vesicles filled by LC3, indicating that recruitment and fusion are not compromised (Fig. 6a). Interestingly, microscopy images revealed abnormal lysosomal clustering close to the perinuclear region after SYT11 knockdown (Fig. 6b), which suggested lysosomal dysfunction. pH and enzymatic activity of the lysosome are well-established markers of its function. Compatible with this assumption, SYT11 knockdown increased the endosomal/lysosomal pH (Fig. 6c; Supplementary Fig. 4f,g) and decreased the activity and the levels of the mature form of cathepsin-L (Fig. 6d,e), which are both pH-dependent^29^,^30^, indicating that SYT11 blocks autophagosome clearance by compromising the lysosomal function.

**Figure 7:**
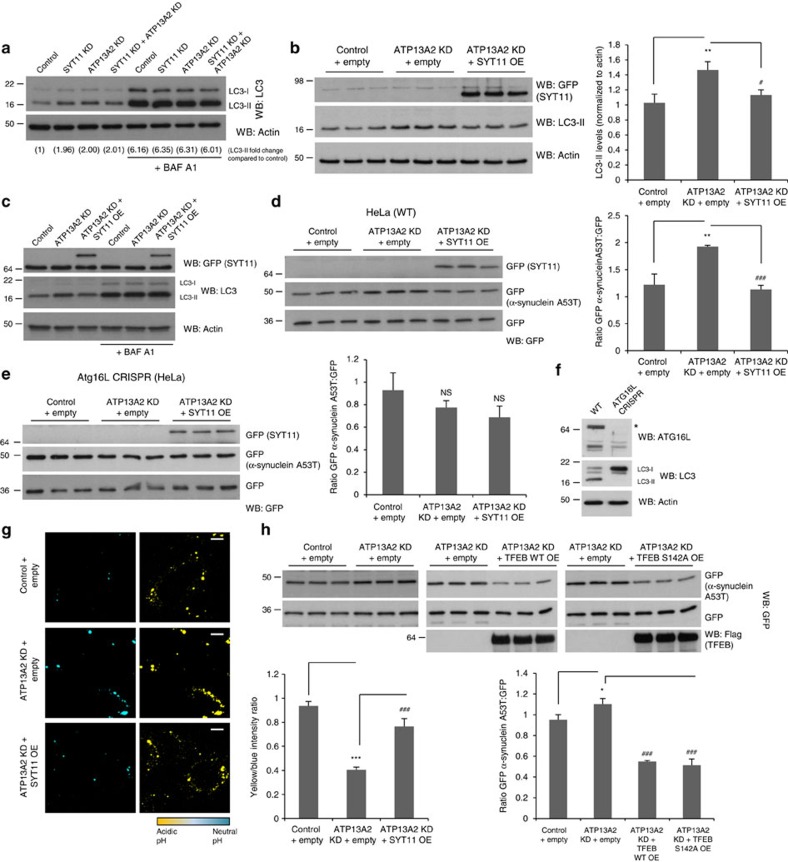
SYT11 overexpression rescues the autophagy blockage phenotype observed under the knockdown of ATP132. (**a**) HeLa cells were transfected with Control, SYT11 siRNA, ATP13A2 siRNA or SYT11 siRNA in combination with ATP13A2 siRNA. Cells were treated with 200 nM bafilomycin A1 (BAF A1) for the last 12 h and lysates were blotted for LC3 and actin. (**b**,**c**) Control and ATP13A2-knockdown HeLa cells were transfected with empty pEGFP or pEGFP-SYT11 in the last 30 h of the experiment (**b**,**c**). Cells were treated with 200 nM BAF A1 for the last 12 h (**c**). Cell lysates were blotted for LC3, GFP (SYT11) and actin. (**d**,**e**) HeLa WT (**d**) or ATG16L CRISPR (**e**) cells were transfected with Control or ATP13A2 siRNA for 5 days. In the last 48 h, cells were transfected with empty vector and pEGFP-SYT11 for 5 h, followed by transfection with empty pEGFP+GFP-α-synuclein A53T. Cells were lysed and blotted for GFP. Representative experiments with triplicates of three independent experiments are shown. Levels of α-synuclein A53T are expressed as a ratio to GFP. (**f**) Lysates obtained from ATG16L-knockout cells produced by CRISPR/Cas9 editing and control HeLa cells were blotted against ATG16L, LC3 and actin in order to validate the knockout efficiency and autophagy competence. (**g**) Control and ATP13A2-knockdown HeLa cells were transfected with empty pcDNA3.1-*myc*/His or pcDNA3.1-SYT11-*myc*/His in the last 30 h of the experiment. Cells were subsequently loaded with LysoSensor Yellow/Blue and analysed by live imaging (scale bar, 10 μM). The graph shows the mean±s.e.m. of the yellow/blue intensity ratio of images obtained from 10 fields. (**h**) HeLa cells were transfected with Control or ATP13A2 siRNA for 5 days. In the last 48 h, cells were transfected with empty pCMV, pCMV TFEB WT or TFEB S142A for 5 h, followed by transfection with empty pEGFP+GFP-α-synuclein A53T. Cells were lysed and blotted for GFP. A representative experiment with triplicates of two independent experiments is shown. Levels of α-synuclein A53T are expressed as a ratio to GFP. All the graphs represent mean±s.d. and statistical significance was determined using two-tailed unpaired Student's *t*-test. *P<0.05; ^**^P<0.01; ^***^P<0.001; ^#^*P*<0.05; ^###^*P*<0.001; NS, not significant.

We were struck that SYT11 and ATP13A2 knockdowns phenocopied each other in many respects. For instance, we observed (as shown by others previously^5^,^8^) that ATP13A2 knockdown impaired autophagosome degradation, both in HeLa cells (Fig. 6f; Supplementary Fig. 4h) and primary mouse neuronal cultures (Fig. 6g), induced lysosomal clustering (Fig. 6h) and increased the lysosomal pH (Fig. 6i). Interestingly, simultaneous knockdown of ATP13A2 and SYT11 did not synergize to additively increase LC3-II levels under basal conditions, as compared with the individual knockdowns (Fig. 7a), reinforcing the idea that these genes might act on autophagy through the same molecular pathway.

### ATP13A2 depletion blocks autophagy via SYT11

To determine whether the autophagy blockage phenotype observed under ATP13A2-depleting conditions was mediated by downregulation of SYT11, we overexpressed SYT11 in ATP13A2-knockdown cells and measured the levels of A53T mutant α-synuclein (the clearance of which is highly dependent on autophagy^31^), in parallel to the levels of LC3-II. SYT11 overexpression was indeed able to reduce the elevated LC3-II levels caused by the knockdown of ATP13A2 to normal levels (Fig. 7b,c), suggesting that SYT11 overexpression re-established the autophagic flux and that ATP13A2-induced blockage of autophagy is triggered by decreased expression of SYT11. Concomitantly, SYT11 overexpression decreased and normalized the levels of α-synuclein A53T that were elevated in ATP13A2-knockdown cells (Fig. 7d), indicating that re-introduction of SYT11 in conditions characterized by decreased levels of ATP13A2 induced increased clearance of α-synuclein by augmenting the autophagic activity. This effect was autophagy-dependent, as these manipulations were ineffective in changing the levels of α-synuclein A53T in autophagy-incompetent cells (ATG16L1-deficient cells, generated by CRISPR/Cas9 editing, that are unable to efficiently produce LC3-II) (Fig. 7e,f). Re-establishment of the autophagic flux by the overexpression of SYT11 under ATP13A2-knockdown conditions was accompanied by normalization of the endo-lysosomal pH (Fig. 7g; Supplementary Fig. 5a) indicating that SYT11 regulates the status of the lysosome. In agreement with our model, we observed that TFEB WT or TFEB S142A mutant overexpression also decreased the levels of α-synuclein A53T in ATP13A2-knockdown cells (Fig. 7h), consistent with increased autophagy-mediated clearance of α-synuclein. The observation that TFEB overexpression not only rescued the levels of α-synuclein A53T under the knockdown of ATP13A2, but also decreased its levels below to the control levels is likely to be explained by the fact that TFEB also induces autophagy^32^. We also observed that SYT11 manipulation itself did not change the levels of TFEB (Supplementary Fig. 2b) and that SYT11 overexpression under ATP13A2-knockdown conditions did not appear to affect the sub-cellular localization of TFEB (Supplementary Fig. 5b), indicating that SYT11 acts indeed downstream of ATP13A2 and TFEB in the network described here. Of interest, ATP13A2 or SYT11 short-term depletion conditions did not significantly affect cellular viability (Supplementary Fig. 1g). However, viability became significantly compromised when cells were insulted with α-synuclein A53T overexpression (Supplementary Fig. 5c), indicating that these two proteins are indeed important for the clearance of α-synuclein and the survival of cells under pathological conditions that are relevant to PD.

**Figure 8:**
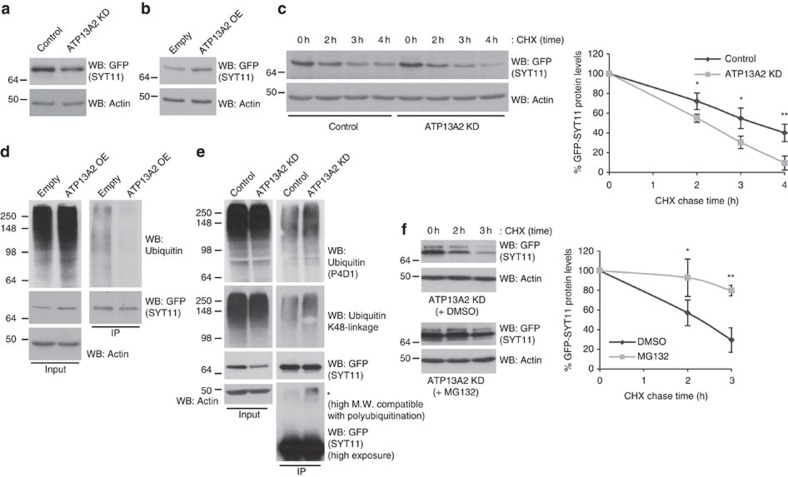
ATP13A2 regulates SYT11 levels by additional post-transcriptional processes. (**a**) Control and ATP13A2-knockdown HeLa cells were transfected with pEGFP-SYT11 for the last 24 h. Cell lysates were blotted against GFP and actin. (**b**) HeLa cells were transfected with empty vector or ATP13A2 WT simultaneously with pEGFP-SYT11 for 24 h. Cell lysates were blotted against GFP and actin. (**c**) Control and ATP13A2-knockdown HeLa cells were transfected with GFP-SYT11 for the last 24 h. In the last 4 h of the experiment, cells were treated with 50 μg ml^−1^ cycloheximide for the indicated time points. Cell lysates were blotted against GFP and actin. Densitometric quantification of the bands was performed and the normalized data (GFP-SYT11 levels to actin levels) of three independent experiments is plotted in the graph. (**d**) HeLa cells were transfected with empty vector or ATP13A2 WT simultaneously with HA-Ubiquitin and GFP-SYT11 for 24 h. Cell lysates were subsequently used for GFP-SYT11 immunoprecipitation and western blotting against GFP, ubiquitin and actin. (**e**) Control and ATP13A2-knockdown HeLa cells were transfected with HA-Ubiquitin and GFP-SYT11 for the last 24 h. Lysates were used for GFP-SYT11 immunoprecipitation and subsequent western blotting against ubiquitin (P4D1 antibody), K48-linkage-specific polyubiquitin conjugates, GFP and actin. (**f**) Control and ATP13A2-knockdown HeLa cells were transfected with GFP-SYT11 for the last 24 h. In the last 5 h, cells were pre-incubated with 10 μM MG132 (or DMSO) and further treated with 50 μg ml^−1^ cycloheximide for the indicated time points. Cell lysates were blotted against GFP and actin. Densitometric quantification of the bands was performed and the normalized data (GFP-SYT11 levels to actin levels) of three independent experiments is plotted in the graph. All the graphs represent mean±s.d. and statistical significance was determined using two-tailed paired Student's *t*-test. *P<0.05; ^**^P<0.01.

Although mTORC1, TFEB and other players downstream of ATP13A2 will have other effects impacting on disease, it is striking that ATP13A2 and SYT11 knockdowns phenocopied each other in multiple respects and that SYT11 robustly rescued the autophagy blockage phenotype caused by ATP13A2 depletion (Fig. 7b,d). This indicated that SYT11 somehow overwhelms all the other autophagy–lysosomal proteins putatively regulated by AP13A2 via TFEB and that SYT11 is indeed a critical downstream target of ATP13A2 in the regulation of autophagy. Therefore, we considered that the regulation of SYT11 levels by ATP13A2 may involve both transcriptional and additional post-transcriptional processes. Indeed, apart from regulating SYT11 transcription, we found that ATP13A2 also regulated SYT11 protein stability. While ATP13A2 knockdown decreased SYT11 protein levels (Fig. 8a), ATP13A2 overexpression had the opposite effect (Fig. 8b). It should be noted that these changes are not due to transcriptional regulation, as SYT11 protein levels were measured by using a tagged-SYT11 expression construct driven by an exogenous promoter. In agreement with the idea that ATP13A2 is likely to regulate SYT11 post-translationally, we observed that ATP13A2 depletion decreased the half-life of SYT11 (Fig. 8c). We also observed that this regulation occurs regardless of whether SYT11 is N- or C-terminal-, GFP- or *c-myc*-tagged (compare Fig. 8a,c and Supplementary Fig. 6a,b). Consistently, ATP13A2 overexpression decreased SYT11 ubiquitination (Fig. 8d), while ATP13A2 knockdown increased SYT11 ubiquitination (Fig. 8e), indicating decreased protein stability. Increased SYT11 ubiquitination under ATP13A2-depleting conditions was also observed using an antibody that specifically recognizes K48-linked polyubiquitin chains (Fig. 8e), which is compatible with proteasome-dependent degradation. In agreement, proteasome inhibition increased the half-life of SYT11 under the knockdown of ATP13A2 (Fig. 8f; Supplementary Fig. 6c), suggesting that ATP13A2 regulates SYT11 ubiquitination and degradation by a mechanism dependent on the proteasome. Overall, the fact that ATP13A2 regulates SYT11, not only at the transcriptional level, but also at the post-translational level provides a plausible explanation for the SYT11-dependency of ATP13A2-mediated autophagy responses.

## Discussion

**Figure 9:**
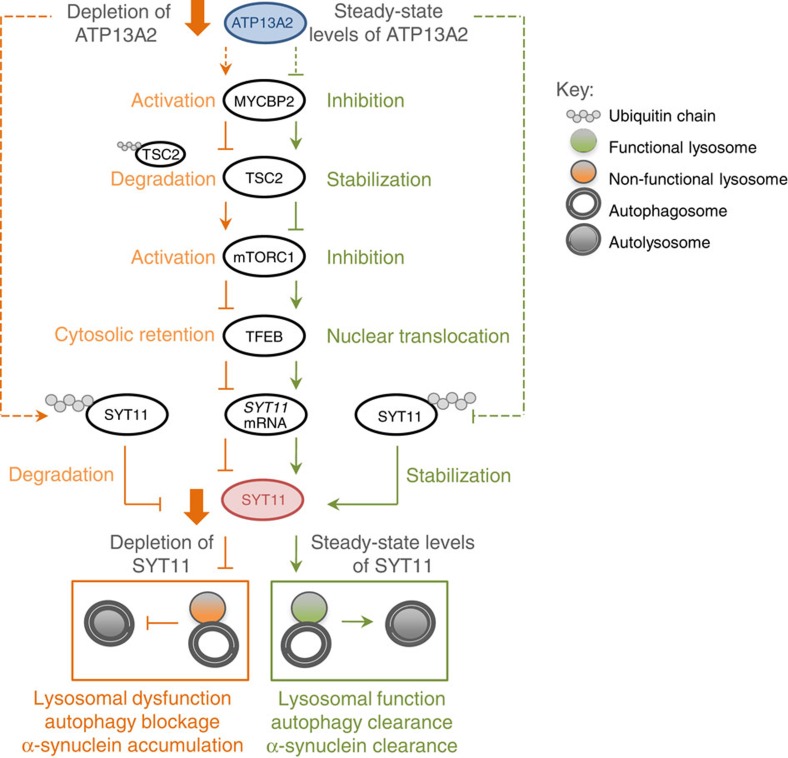
Model proposed on how ATP13A2 and SYT11 establish a common network that regulates the autophagy–lysosomal pathway. Orange arrows identify the sequence of events that occur when ATP13A2 is depleted from cells, which in turn leads to depletion of SYT11, while green edges identify the sequence of events when ATP13A2 and SYT11 remain under steady-state conditions. Overall, we propose that ATP13A2 depletion decreases TSC2 levels, by a mechanism dependent on MYCBP2-induced ubiquitination, which in turn induces activation of mTORC1 and decreased TFEB-mediated transcription of *SYT11*. In parallel, ATP13A2 depletion induces SYT11 ubiquitination and degradation. Both events contribute to a decrease of SYT11 levels, which induces lysosomal dysfunction, autophagy blockage and increased accumulation of α-synuclein A53T.

Lysosomal and autophagy impairment appear to be hallmarks of PD; however the mechanisms involved in these dysfunctions are still poorly understood. Here we describe a new functional network established by ATP13A2 and SYT11, providing some new understanding into the putative mechanisms underlying the lysosomal and autophagy impairment phenotypes observed in some models of the disease. While our data suggest that ATP13A2 and SYT11 are in the same pathway, this does not mean they act in the same functional complex. For instance, these two proteins certainly have very distinct roles. Accordingly, we found for the first time that ATP13A2 downregulation decreases TSC2 levels, by a mechanism dependent on MYCBP2-induced ubiquitination, which in turn activates mTORC1, inhibits TFEB translocation to the nucleus and compromises TFEB-mediated transcription of *SYT11*. We observed that ATP13A2 also promotes ubiquitination of SYT11 and its degradation in a proteasome-dependent manner. Both mechanisms account for a pronounced decrease of SYT11 levels in cells, which in turn causes lysosomal dysfunction, impairment of autophagosomes degradation and α-synuclein A53T accumulation (Fig. 9), all being PD hallmarks.

Although we were able to show that ATP13A2, MYCBP2 and TSC2 co-immunoprecipitate together (confirming previous results^15^,^25^), we did not observe significant changes in the physical interaction between MYCBP2 and TSC2 when ATP13A2 was depleted, suggesting that ATP13A2 is likely to act as a positive regulator of TSC2 through inactivation of MYCBP2 E3-ligase activity towards TSC2, rather than by affecting the affinity between the two proteins. For instance, ATP13A2 can regulate zinc homeostasis^33^,^34^, and MYCBP2 is an E3 ligase with zinc requirements due to the presence of B-box-type zinc finger and RING-type zinc finger domains in its sequence (UniProt accession number O75592). Therefore, one possibility is that ATP13A2 indirectly regulates MYCBP2 activity by controlling zinc availability and/or establishing zinc microenvironments in the vicinity of the complex.

The regulation of SYT11 ubiquitination by ATP13A2 observed here also requires further understanding. SYT11 was previously shown to be ubiquitinated by Parkin^35^, which is a RING-type E3 ligase that also has zinc requirements (UniProt accession number O60260); however, HeLa cells have little endogenous Parkin expression, compared with neuronal cells^36^. Considering the observation that ATP13A2 regulates SYT11 levels not only in neurons but also in HeLa cells, we speculate that: (1) a little Parkin expression is enough to sustain SYT11 ubiquitination in HeLa cells, or (2) ATP13A2 induces SYT11 ubiquitination by signalling through a Parkin-independent mechanism (even in neurons) or (3) another ubiquitin ligase can compensate for the lack of Parkin in non-neuronal cells.

It is interesting to note the existence of mechanistic parallels between various pathways known to be impaired in PD. Mitophagy is indeed one of these pathways, where PINK-1-dependent recruitment of Parkin to damaged mitochondria induces ubiquitination of mitochondrial membrane proteins (for example, mitofusin-2 or MFN2), which serves as a signal to recruit the autophagy machinery and sustain mitochondria degradation^37^. On the other hand, the pathway described here involves regulation of the lysosomal and autophagic activities by a mechanism where ATP13A2 controls the mRNA and protein levels of SYT11 (by several ubiquitination-dependent events), which is likely to regulate, in turn, the shuttling of key proteins and enzymes to the lysosome necessary for its normal function. Faults in these pathways are therefore expected to culminate in the accumulation of autophagy substrates such as damaged/aged mitochondria and α-synuclein aggregates, which are known to enhance susceptibility to neuronal cell death. Interestingly, ATP13A2 depletion was observed to compromise mitochondrial function and clearance due to reduced autophagic flux^38^, emphasizing the possible crosstalk between the PINK1/Parkin and ATP13A2/SYT11 pathways in PD.

Despite the fact that TFEB has been shown to have a key role in the modulation of several lysosome-related diseases^3^,^39^,^40^, no *TFEB* disease-associated variants have been found so far. However, the fact that ATP13A2 appears in this work as a new TFEB regulator provides a hypothetical and indirect pathophysiological context for the plausible contribution of TFEB to the development of PD and, eventually, other neurodegenerative disorders.

mTORC1 is a key regulator of protein translation, whose activation is canonically associated with increased protein synthesis and levels, and also regulates specific cellular pathways by controlling complex transcriptional programs. A genomic approach to identify mTORC1-regulated transcripts surprisingly identified not only mTORC1-induced genes but also mTORC1-repressed genes^41^. In addition, mTORC1 activation was also shown to positively control some cytoplasmic 5′-3′ mRNA degradation mechanisms^42^ and inhibit synthesis of certain transcripts by inactivation of specific transcription factors, such as TFEB in mammals^20^, and Rim15, Msn2/4 and Gln3 in yeast^42^. This shows that transcriptional regulation by mTORC1 is not as linear and simple as once thought, and that mTORC1 inhibition by rapamycin increases the mRNA levels of certain genes, like *SYT11* in the specific case of this work. Consistent with our model, rapamycin was interestingly observed by others to rescue autophagic defects in ATP13A2-depleted cells^38^. However, these autophagic defects were attributed to early autophagy inhibition rather than to autophagy late blockage^38^. Although we do not exclude the possibility of simultaneous early inhibition of autophagy (as mTORC1, a well-known autophagy inhibitor, is activated by the knockdown of ATP13A2) and autophagy late blockage, the authors measured the autophagic flux by treating cells with a non-saturating concentration (5 nM) of bafilomycin A1, which is not sufficient to robustly block autophagosome–lysosome fusion and accurately measure autophagic flux^43^. In addition, numbers of autophagosomes and autolysosomes were the only RFP-GFP-LC3 assay readout considered; size of vesicles is also an important readout for identifying autophagy late blockers, as large size and intensity might mask the total number of vesicles, which cannot fuse and/or be degraded in this scenario.

The concomitant occurrence of mTORC1 hyperactivation and lysosomal dysfunction resulting from ATP13A2 knockdown may be surprising, as integrity of the lysosomal proton-pump ATPase has been widely proposed to be necessary for mTORC1 activation, in conjugation with Ragulator and Rag GTPases in the lysosomal membrane^44^. However, several independent groups have shown that abnormal lysosomal pH can increase activation of mTORC1 in some cell lines, at least^45^,^46^. In addition, FCCP, an ionophore capable of dissipating the lysosomal proton gradient without interfering with the V-ATPase, does not appear to abrogate amino-acid-induced binding of Raptor (the Regulatory-Associated Protein of mTORC1) to RagB in lysosomes, suggesting that maintenance of lysosomal proton gradient by V-ATPase is dispensable for mTORC1 activation in lysosomes^47^ and that mTORC1 can be activated in the context of increased lysosomal pH. Thus, our data are not inconsistent with the literature, since mTORC1 activation/hyperactivation does not require full lysosomal function.

Although this is the first study to ascribe a role to SYT11 in the regulation of the autophagy–lysosome pathway, this is not of complete surprise. SYTs constitute a family of proteins that are generally involved in calcium-dependent vesicular fusion, trafficking and exocytosis, having a major role in the regulation of neurotransmitter release and hormone secretion by virtue of the calcium-binding affinity of their C2 motifs. However, SYT11 is unexpectedly thought to be, at least in part, calcium-insensitive^48^,^49^,^50^, suggesting specific and unpredicted roles and further levels of regulation. (As an example, SYT11 has been shown to function as a negative regulator of endocytosis, controlling vesicle retrieval, while Ca^2+^-binding SYTs are normally seen as positive regulators of endocytosis^51^). Therefore, we postulate that SYT11 is likely to affect autophagy either by inhibiting the fusion between autophagosomes and lysosomes (as SYT proteins are canonically implicated in vesicular fusion events by assisting SNAREs) or by directly regulating the activity of lysosomes (for example, by controlling the delivery of hydrolases or V-ATPase components to the lysosome from the Golgi compartment). This last hypothesis sounds particularly plausible not only because coalescence of LAMP-1-positive and LC3-positive vesicles did not appear to be affected by SYT11 depletion but also because the lysosomal pH and enzymatic activity were both observed to be disturbed under SYT11-depleting conditions. In addition, other SYTs are implicated in the recruitment of LAMP1 and V-ATPase to phagosomes^52^,^53^, indicating that SYT11 depletion might impact the function of degradative vesicular compartments within the cell.

In conclusion, our data shows that ATP13A2 regulates SYT11 at both transcriptional and post-translational levels. We found that mRNA expression of *SYT11* is regulated by the TSC2/mTORC1/TFEB transcriptional axis, while SYT11 protein levels are regulated by ubiquitination and proteasomal-dependent degradation mechanisms. Defects in this pathway are associated with lysosomal dysfunction, autophagy impairment and accumulation of α-synuclein A53T, which are plausible contributors to PD pathogenesis. Therefore, while other studies are required to elucidate further aspects of the pathway and the disease, we believe this work fosters solid ground for future investigations.

## Methods

### Cell lines

HeLa, SH-SY5Y, SK-N-SH (from ATCC) and GFP-P62 HEK293 Flp-In T-REx cells (gift from Dr Terje Johansen)^54^ were maintained at 37 °C and 5% CO_2_ and cultured in Dulbecco's modified Eagle's medium (DMEM) (4.5 mg  l^−1^ of glucose) supplemented with 10% FBS, 2 mM L-glutamine and 100 U ml^−1^ penicillin and 100 μg ml^−1^ streptomycin (all the components were obtained from Sigma Aldrich). HeLa cells stably expressing mRFP-EGFP-LC3 were cultured also in the presence of geneticin at a concentration of 500 μg ml^−1^, while the GFP-P62 HEK293 Flp-In T-REx cells were maintained in the presence of 7.5 μg ml^−1^ blasticidin and 100 μg ml^−1^ hygromycin. All the lines were regularly tested for mycoplasma contamination.

### Reagents

The following primary antibodies have been used in this work: mouse anti-ATP13A2 (#110-41486, Novus Biologicals); mouse anti-cathepsin L clone 22 (#611084, BD Transduction Laboratories; immunoblotting concentration 1:300); mouse anti-Flag M2 (#F3165, Sigma Aldrich); mouse anti-GAPDH clone 6C5 (#ab8245, Abcam); mouse anti-GFP (#632375; Clontech); mouse anti-turboGFP clone OTI2H8 (#TA150041; Origene); mouse anti-HA.11 clone 16B12 (#MMS-101P, Covance), mouse anti-LAMP1 clone H4A3 (obtained from Developmental Studies Hybridoma Bank, University of Iowa); mouse anti-LC3 clone 5F10 (#0231, Nanotools); mouse anti-*c-myc* clone 9E10 (#11667203001; Roche); mouse anti-V5 (#46-0705, Invitrogen/Life Technologies); mouse anti-TFEB (#MBS120432, MyBioSource); mouse anti-ubiquitin P4D1 (#3936, Cell Signaling); rabbit anti-actin (#A2066, Sigma Aldrich); rabbit anti-ATG16L (#PM040, MBL); rabbit anti-ATP13A2 (#5879, Cell Signaling); rabbit anti-GFP (#632592, Clontech); rabbit anti-LAMP1 (#9091, Cell Signaling); rabbit anti-LC3 (#NB 100-2220, Novus Biologicals); rabbit anti-phosphoSer2481 mTOR (#2974, Cell Signaling); rabbit anti-phosphoThr389 p70S6kinase (#9234) and anti-total p70S6kinase (#9202) (Cell Signaling); rabbit anti-MYCBP2 (#ab86078, Abcam); rabbit anti-TFEB (#4240, Cell Signaling); rabbit anti-phosphoSer1387-TSC2 (#5584), anti-phosphoThr1462-TSC2 (#3617) and anti-total TSC2 (#4308) (Cell Signaling); rabbit anti-K48-linkage-specific polyubiquitin (#4289, Cell Signaling); rabbit anti-ZNF306/ ZKSCAN3 (#AV33609; Sigma); goat anti-Lamin B M20 (#sc-6217, Santa Cruz Biotechnology); goat anti-SYT11 N-15 (#sc-162280, Santa Cruz Biotechnology); goat anti-TFEB (#ab2636; Abcam); anti-mouse (#NA931V) and anti-rabbit (#NA934V) horseradish peroxidise (HRP)-conjugated secondary antibodies (GE Healthcare); anti-goat horseradish peroxidise (HRP)-conjugated secondary antibody (#611620, Invitrogen/Life Technologies); Alexa Fluor 488- (#A11001) and Alexa Fluor 568- (#A11004) conjugated goat anti-mouse (Invitrogen/Life Technologies); Alexa Fluor 488- (#A11008) and Alexa Fluor 568- (#A11036) conjugated goat anti-rabbit secondary antibodies (Invitrogen/Life Technologies). For western blotting, all the primary antibodies were used at a dilution of 1:1,000 (overnight incubation at 4 °C), unless otherwise stated, and the secondary antibodies used at a dilution between 1:2,000 and 1:5,000 (1 h of incubation at room temperature), while for immunofluorescence primary antibodies were used at a range of 1:50 to 1:200 (overnight incubation at 4 °C) and secondary antibodies used at a dilution of 1:400 (1 h of incubation at room temperature in the dark). The following constructs were also used in this work: empty pEFGP; pEGFP-LC3; pEGFP-SYT11 (kindly provided by Dr Stefan Pulst)^35^; pCMV6-SYT11-TurboGFP (RG205445, Origene); pcDNA3.1-*myc*-His (Invitrogen); pcDNA3.1-SYT11/*myc*-His; *SYT11* promoter-GLuc/SEAP pEWZX-PG04 (HPRM12158-PG04, GeneCopoeia); pEGFP-α-synuclein A53T; pcDNA3.1; pcDNA3.1 ATP13A2 WT, pcDNA3.1 ATP13A2 delC (3057 delC) mutant and pcDNA3.1 ATP13A2 i16 (1632_1653dup22) mutant (all V5-tagged) (kindly provided by Dr Christian Kubisch)^7^; empty pCMV; pCMV TFEB WT-3xFlag and pCMV TFEB S142A-3xFlag constitutively active mutant (kindly provided by Dr Andrea Ballabio)^20^; pcDNA3 Flag-TSC2 (#14129, Addgene)^55^; pcDNA3.1 HA-ubiquitin; pSpCas9n(BB)-2A-GFP (PX461) vector (#48410, Addgene). The following inhibitors were used in this work: Bafilomycin A1 (Millipore); Torin-1 (Millipore); Cycloheximide (Sigma Aldrich); MG132 (Sigma Aldrich); Rapamycin (Sigma Aldrich). Pre-designed siRNAs (SMARTpool and/or set of deconvoluted oligos ON-TARGETplus>Non-targeting Control #D-001810-10; SYT11 #L-018292, ATP13A2 #L-008601, MYCBP2 #L-006951; TFEB #L-009798; ZKSCAN3 #L-014607) were obtained from Dharmacon—Thermo Scientific. Pre-designed pLKO.1 shRNAs vectors from The RNAi Consortium (TRC) (empty vector control—#RHS4080, Dharmacon; eGFP shRNA control—#SHC005, Sigma Aldrich; mouse SYT11—#RMM4534-EG229521, Thermo Scientific; mouse ATP13A2 shRNAs—#SHCLNG-NM_029097, Sigma Aldrich) were also used in this work. Although all the shRNAs provided by TRC were validated, some were selected and used to perform most of the experiments (SYT11: TRCN0000106535 (#A) and TRCN0000093552 (#B); ATP13A2: TRCN0000101717 (#A), TRCN0000101718 and TRCN0000101719 (#B)).

### Mutagenesis and sub-cloning

Mutagenesis of the human WT *SYT11* promoter-GLuc-SEAP vector (GeneCopoeia) (CLEAR-Site 2 in *SYT11* promoter; CTCAAGTGAT>CAAAAGAAAT) was generated with QuikChange Multi Site-Directed Mutagenesis Kit (Agilent Stratagene), according to manufacturer's instructions, using the set of primers listed in Supplementary Table 1. *DPN I* digestion was performed after PCR and XL-10 gold-competent cells were transformed. After sequencing, *SYT11* mutant promoter from a positive clone was subcloned into the original vector using *EcoR I* and *Hind III* restriction enzymes (New England BioLabs Inc.) followed by ligation using T4 DNA ligase (New England BioLabs Inc.). The final vector was confirmed by sequencing. For the production of pcDNA3.1-human SYT11/*myc*-His, human SYT11 complementary DNA was subcloned into pcDNA3.1/*myc*-His (Invitrogen) using *EcoR I* and *Xho I* (New England BioLabs Inc.) for restriction and T4 DNA ligase (New England BioLabs Inc.) for ligation. Efficient subcloning and protein expression were confirmed by sequencing and western blotting against *c-myc*, respectively.

### Isolation and culture of primary cortical neurons

All mouse experiments were performed with personal and project licences granted by the UK Home Office and with the approval of the University of Cambridge committee for animal studies. Primary cortical neurons were isolated from C57BL/6 mice (Jackson Laboratories) embryos at E16.5. Briefly, brains were harvested and placed in PBS/glucose where the meninges were removed and the cerebral cortices were dissected. After mechanical dissociation using sterile micropipette tips, dissociated neurons were resuspended in PBS/glucose and collected by centrifugation. Viable cells were seeded on poly-ornithine-coated 6- or 12-multiwell plates. Cells were cultured in Neurobasal medium supplemented with 2 mM glutamine, 200 mM B27 supplement and 1% Penicillin–Streptomycin at 37 °C in a humidified incubator with 5% CO_2_. One half of the culture medium was changed every 2 days until treatment/infection. After 5 days of *in vitro* culturing, differentiated neurons were infected with lentiviral particles^56^.

### Generation of CRISPR/Cas9 HeLa cell line for ATG16L1

HeLa CRISPR/Cas9 ATG16L1 knockout cell line was generated using double nicking to minimize off target cleavage as previously described^57^,^58^. Briefly, pairs of guided RNAs (gRNAs) nicking the first exon of ATG16L1 (192-211 and 146-166) were designed using the CRISPR design tool available at Zhang Lab website (http://www.genome-engineering.org/crispr/). gRNAs with overhangs were annealed (without phosphorylation) and cloned into *BbsI*-digested pSpCas9n(BB)-2A-GFP (PX461) vector (#48140, Addgene) as described before^58^. Ligation was performed using T4 DNA ligase based on ‘ELAN' method^59^. HeLa cells were subsequently transfected with pairs of gRNAs cloned into PX461 or with empty PX461. Two days after transfection, GFP-positive cells were single-cell sorted into 96-well plates using fluorescent activated cell sorting (FACS). Clones were expanded and those where almost undetectable levels of ATG16L1 and autophagy activity were observed were selected. While it should be noticed that residual ATG16L1 levels might still be observed, for the purpose of our experiments these are autophagy-incompetent clones.

### GFP-P62 clearance assay

GFP-P62 HEK293 Flp-In T-REx cells^54^ (gift from Dr Terje Johansen) were used to evaluate P62 clearance, an indicator of the autophagic flux. GFP-P62 expression under the control of the CMV/TetO_2_ promoter was induced by adding 1 μg ml^−1^ tetracycline to the culture medium for 24 h. Cells were then washed twice with PBS to switch off the transgene expression and kept in full medium without tetracycline for 18 h. P62 clearance was determined by blotting the cell lysates against GFP and performing T18/T0 (h) ratio of the densitometry measurements of GFP-P62 bands.

### Transfection

Trans IT-2020 reagent (Mirus) was used for DNA transfections, while Lipofectamine 2000 (Invitrogen/Life Technologies) was used for siRNA transfections, according to the manufacturer's instructions. After transfection, cells were maintained in full medium. For knockdown experiments, unless otherwise specified in figure legends, cells were transfected with 50 nM siRNA followed by another 50 nM siRNA transfection after 48 h. Cells were split once in between both transfections and harvested after 4 days of transfection.

### α-synuclein A53T assay

Cells were transfected with 1.5 μg of pEGFP-α-synuclein A53T and 0.5 μg of empty pEGFP per well of a six-well plate. Forty-eight hours after transfection, cells were lysed and used to analyse GFP-α-synuclein A53T and GFP levels by western blotting. Ratio between both signals was calculated.

### shRNA lentivirus production and infection

shRNA lentiviral particles were produced and transduced following The RNAi Consortium (TRC) protocols. Briefly, HEK-293T packaging cells growing in 100 mm dishes were transfected at 50–60% of confluence with a mix of 2.5 μg psPAX2 vector (packaging vector), 270 ng pMD2.G vector (envelope vector) and 2.7 μg hairpin-pLKO.1 vector. TransIT-LT1 (Mirus) was used as transfection reagent according to the manufacturer's instructions. After transfection, cells were cultured in high-serum medium. Cell culture medium was harvested 40 h later and replaced by high-serum medium; this step was repeated two to three times for intervals of 24 h. Viral preps were then concentrated by centrifugation at 160,100*g* for 90 min. Depending on the cell type, different viral titres were added to the cells in the presence of 4 μg ml^−1^ polybrene (Sigma Aldrich) and were incubated overnight. 24 h later, medium was replaced by full medium and cells were further incubated for an additional 3–4 days before testing the knockdown effects.

### Western blot

Cells were washed with ice-cold PBS and directly lysed with 2 × Laemmli buffer and boiled at 95 °C for 5 min or lysed with RIPA buffer (50 mM Tris-HCl pH 7.4, 150 mM NaCl, 1% NP-40, 0.5% sodium deoxycholate monohydrate, 0.1% SDS, supplemented with protease and phosphatase inhibitors cocktails (Roche)). When lysed in RIPA buffer, cells were incubated on ice for 30 min, centrifuged at 16,100 *g* for 10 min and protein concentration of supernatants was determined using a Bradford assay kit (Bio-Rad). Lysates were then denatured with 2 × Laemmli buffer and boiled at 95 °C for 5 min. Cell extracts were resolved by SDS–PAGE and transferred electrophoretically onto polyvinylidene fluoride (PVDF) membranes. The membranes were blocked with 5% nonfat milk in PBS-T (PBS, 0.2% Tween 20) and probed for the proteins of interest, using specific primary and HRP-conjugated secondary antibodies, according to the respective manufacturer's instructions. Immunoreactive bands were visualized with an ECL system (Pierce, Thermo Scientific). For detection of very high molecular weight proteins (for example, MYCBP2), precast NuPAGE Novex 3-8% Tris-Acetate protein gels (Thermo Fisher Scientific) were used according to the manufacturer's instructions. Densitometric analysis on the immunoblots was performed using Image J. Full blot images are shown in Supplementary Fig. 7.

### Cytosolic/nuclear fractionation

Cells were washed twice with ice-cold PBS and lysed with four packed cell volumes of Buffer A (10 mM HEPES, 10 mM KCl, 0.1 mM EDTA, 0.4% NP-40, 1 mM DTT and protease/phosphatase inhibitors cocktail) and incubated on ice for 30 min. Cell lysates were centrifuged at 16,100*g* at 4 °C for 10 min. Supernatants containing cytosolic proteins were harvested and nuclear pellets were resuspended in 3.5 packed cell volumes of Buffer B (20 mM HEPES, 0.4 M NaCl, 1 mM EDTA, 10% glycerol, 1 mM DTT and 1 × protease inhibitor cocktail) and incubated for 1 h on ice. After centrifugation at 16,100*g* for 10 min at 4 °C, supernatants containing the nuclear proteins were collected. Both cytosolic and nuclear fractions were denatured and used to perform immunoblot against TFEB, ZKSCAN3, Lamin-B and GAPDH. Protein concentration was determined using the Bradford assay (Bio-Rad).

### Immunoprecipitation and mass spectrometry

Cells in 60 mm dishes were washed twice with PBS and collected in ice-cold PBS. Pellets were resuspended in 200 μl of lysis buffer (20 mM Tris-HCl, pH 7.2, 150 mM NaCl, 2 mM MgCl_2_, 0.5% NP-40, protease/phosphatase inhibitors cocktails, and further supplemented with 1 mM PMSF, 10 mM iodoacetamide for the ubiquitination assays). Lysates were incubated on ice for 30 min and cleared by centrifugation at 16,100*g* for 10 min. Supernatants were transferred to new tubes; 1/10 of the sample was kept as whole cell lysate/input control, while the remaining lysate was overnight incubated with primary antibodies at 4 °C with gentle agitation. Thereafter, Dynabeads-protein G (Life Technologies) or Protein A Sepharose beads (for TSC2 ubiquitination assays; GE Healthcare) were added to the samples and incubations proceeded at 4 °C for 2 h. Beads were washed three times with lysis buffer and the immunoprecipitated proteins were eluted and denatured with 2 × Laemmli buffer and boiled at 100 °C. Samples were loaded on SDS–PAGE and western blot analyses were performed. Considering that the levels of TSC2 and SYT11 are affected by the genetic manipulations described in the figures, we adjusted the immunoprecipitations loading volumes, keeping the levels of the immunoprecipitated protein even (using the levels of the respective protein in the inputs as reference), to assess protein ubiquitination.

For mass spectrometry, samples were resolved ∼1 cm into a pre-cast SDS–polyacrylamide gel, the entire lane excised and cut into four equal slices. Proteins were reduced and alkylated, and subsequently digested in-gel using trypsin. The resulting peptides were analysed by LC-MS/MS in a DDA manner using a Q Exactive (Thermo Scientific) coupled to a RSLC3000 nanoUPLC (Thermo Scientific). Raw files were converted to mzML using MSconvert (ProteoWizard) and submitted to MASCOT 2.3.0 to search a human Uniprot database (20,264 entries, downloaded 09/06/14). Peptide and protein validation was performed using Scaffold 4.3.2. Likely hits required a minimum of 95% probability and proteins a minimum of 90% probability and two peptides.

### Immunofluorescence and microscopy

Cells growing in coverslips were fixed with 4% paraformaldehyde (unless otherwise stated). The fixed samples were permeabilized with 0.1% Triton X-100 (v/v) for 10 min and blocked with 10% goat serum or 2% BSA in PBS for 1 h prior to overnight incubation with primary antibodies (concentration ranging from 1:50 to 1:200). The samples were then washed three times with PBS and incubated with 1:400 Alexa Fluor 488- or Alexa Fluor 568-conjugated goat anti-mouse or anti-rabbit secondary antibodies (Invitrogen/Life Technologies, Carlsbad, CA, USA) for 1 h at room temperature. The samples were then washed three times with PBS and once with water and mounted with ProLong Antifade reagent containing DAPI (Invitrogen/Life Technologies). The samples were imaged by LSM170 confocal microscopy (Zeiss). ImageJ and Photoshop (Adobe) were used for further analysis and processing of confocal images. Automated counting and analysis of area and intensity of autophagosomes and autolysosomes using a HeLa stable cell line expressing mRFP-EGFP-LC3 was performed using an ArrayScan VTi HCS system (Cellomics).

### Proximity ligation assay

The proximity ligation assay kit was obtained from Sigma Aldrich and used according to manufacturer's instructions. Briefly, cells growing in coverslips were fixed with 4% paraformaldehyde in PBS, permeabilized with 0.1% Triton X-100 in PBS and blocked with 2% BSA in PBS. Subsequently, cells were incubated with primary antibodies (1 h at room temperature), which was followed by incubation of cells with secondary antibodies conjugated to oligonucleotide primers, also designated proximity ligation assay probes MINUS and PLUS (1 h at 37 °C). The primers were then ligated (30 min at 37 °C) and then rolling circle amplification (100 min at 37 °C) was used to create a reaction product that is observable by microscopy due to hybridization of fluorescently labelled nucleotides. Successful production of a DNA product requires that the primary antibodies bind their respective antigens and reside within 40 nm of each other. Coverslips were mounted on slides and imaged by epifluorescence microscopy.

### Cathepsin-L activity

Briefly, cells were lysed in cold CL lysis buffer (Abcam) and incubated on ice for 10 min. The lysates were cleared by centrifugation at 16,100*g* for 5 min at 4 °C. To 40–50 μg of protein (in 50 μl), we added 50 μl of CL reaction buffer (Abcam) and 2 μl of 10 mM Ac-FR-AFC fluorogenic substrate (Abcam). Samples were incubated for 2 h at 37 °C and fluorescence was measured using a plate reader and the following settings: *λ*_ex_ 400 nm; *λ*_em_ 505 nm. Fluorescence was measured using a Versamax Tunable microplate reader (Molecular Devices).

### Cell viability assay

CellTiter-Glo Luminescent Cell Viability assay (Promega) was used to assess cell viability, according to manufacturer's instructions. This assay determines the number of viable cells in culture by quantifying the ATP present, which reflects the number of metabolically active cells. Briefly, cells seeded in 96-well plates were incubated with a volume of CellTiter-Glo reagent (CellTiter-Glo substrate previously reconstituted in CellTiter-Glo buffer) equal to the volume of cell culture medium present in each well. Contents were mixed for 2 min on an orbital shaker to induce cell lysis and subsequently incubated at room temperature for 10 min to stabilize luminescent signal. Homogenates were then transferred to a white opaque 96-well plate and luminescence was measured with an integration time of 0.5 s per well using GLOMAX 96-microplate luminometer (Promega). Results were normalized towards protein concentration.

### Annexin V-FITC apoptosis detection assay

Cells were gently trypsinized and washed once with serum-containing media. 2.5 × 10^5^ cells were collected by centrifugation and re-suspended in 500 μl of binding buffer. Annexin V-FITC (5 μl) and propidium iodide (PI; 5 μl) were then added to the cell suspension and incubation proceeded for 5 min at room temperature in the dark (Annexin V-FITC/PI Kit, Abcam). Annexin V-FITC binding and PI staining were analysed by flow cytometry using FL1 (ex=488 nm; em=530 nm) and FL2 (ex=488 nm; em=585 nm) detectors, respectively, and appropriate compensation settings (FACSCalibur). Annexin V-FITC(-)/PI(-) represent viable cells, Annexin V-FITC(+)/PI(-) represent early apoptotic cells and Annexin V-FITC(±)/PI(+) represent late apoptotic/necrotic cells.

### Luciferase reporter assay

Cell culture medium from cells transfected with human WT *SYT11* promoter-GLuc-SEAP (pEZX-PG04; GeneCopoeia) or CLEAR-site 2 mutated *SYT11* promoter-GLuc-SEAP vectors for 48 h was collected. Secrete-Pair Dual Luminescence Assay Kit (GeneCopoeia) was used, according to manufacturer's instructions, to analyse the activities of *Gaussia* Luciferase (GLuc) and secreted alkaline phosphatase (SEAP) in cell culture medium. Both GLuc and SEAP are secreted reporter proteins. *SYT11* promoter controls GLuc reporter gene expression, while SEAP is controlled by a cytomegalovirus (CMV) promoter. SEAP expression was used as a normalization factor (internal standard control). Briefly, 10 μl of culture medium samples were pipetted into a 96-well white opaque plate (samples need to be heated at 65 °C for 15 min when measuring SEAP expression), which were supplemented with 100 μl of GLuc assay working solution (buffer GL-S in water+substrate GL) or SEAP assay working solution (buffer AP in water+substrate AP). Homogenates were incubated at room temperature for 1 min (GLuc) or 5 min (SEAP) and luminescence was subsequently measured (GLOMAX 96-microplate luminometer, Promega). The ratio of luminescence intensities (RLU, relative light unit) of GLuc over SEAP was calculated for each sample.

### Endosomal/lysosomal pH measurement

Endosomal/lysosomal pH was determined using the pH-sensitive LysoSensor Green DND-189 dye (Invitrogen/Life Technologies) at a working concentration of 1 μM, according to the manufacturer's instructions. Briefly, cells were incubated with 1 μM LysoSensor Green DND-189 in normal cell culture medium for 1 h at 37 °C. For the calibration curve, cells were incubated with pH-fixed MES buffer (5 mM glucose, 20 mM MES, 1 mM CaCl_2_, 1 mM MgCl_2_, 130 mM NaCl and 10 mM KCl; pH 3.0, 4.0, 5.0 and 6.0) in the presence of 10 μM nigericin (Sigma Aldrich) and 10 μM monensin (Sigma Aldrich). Fluorescence was measured with a Versamax Tunable microplate reader (Molecular Devices) (*λ*_ex_ 443 nm, *λ*_em_ 505 nm). Alternatively, pH determination of acidic organelles was assessed using the ratiometric LysoSensor Yellow/Blue DND-160 (Invitrogen/Life Technologies) probe at a final concentration of 1 μM, which produces blue fluorescence in a neutral environment but shifts to yellow fluorescence in more acidic compartments (pKa≈4.2), according to the manufacturer's instruction. Briefly, 80–90% confluent cells seeded on a glass bottom culture dish (MatTek Corporation) were loaded with the LysoSensor tracer for 30 min at 37 °C, washed twice with medium and live imaged immediately at 37 °C. Cells were excited at 365 nm and images were taken at 450 and 510 nm of emission, respectively. ImageJ was used for analysis^60^.

### Real-time PCR

Total RNA was purified using TRIzol (Invitrogen/Life Technologies) or using RNeasy Mini kit columns (Qiagen), according to the manufacturer's instructions. Total RNA samples were then treated with RNase-free DNase I (Invitrogen/Life Technologies) to avoid genomic DNA contamination. SuperScript III First Strand Synthesis kit (Invitrogen/Life Technologies) was used to synthesize the first cDNA strand according to the manufacturer's instructions. For real-time PCR, SYBR Green PCR master mix reagent (Invitrogen/Life Technologies) was used and cDNA amplification was performed according to the manufacturer's protocols. GAPDH was used as housekeeping gene and the set of primers listed in Supplementary Table 1 were used for single amplification reactions. All the real-time PCR analyses were conducted on a 7900HT Fast Real-Time PCR System (Applied Biosystems) and relative quantitation was performed based on the ΔΔCT method. Results represent fold induction compared with the respective control.

### Chromatin immunoprecipitation

HeLa cells (1 × 10^8^ per condition) were incubated with 1% formaldehyde (cross-linker) in cell culture medium for 10 min at room temperature. To stop the crosslinking, cells were subsequently treated with 0.215 M Glycine for 5 min at room temperature and then washed twice with PBS. Cells were lysed in buffer A (10 mM Tris pH 8.0, 10 mM NaCl, 0.2% NP40 supplemented with 10 mM NaBu and protease/phosphatase inhibitors cocktail) for 10 min on ice. The nuclei were recovered by centrifuging the lysates at 3,000*g* for 5 min at 4 °C, resuspended in buffer B (50 mM Tris pH 8.0, 10 mM EDTA, 1% SDS supplemented with 10 mM NaBu and protease/phosphatase inhibitors cocktail) and incubated for 10 min on ice. Samples were then diluted 2 × in buffer C (20 mM Tris pH 8.0, 2 mM EDTA, 150 mM NaCl, 1% Triton X100, 0.01% SDS supplemented with 10 mM NaBu and protease/phosphatase inhibitors cocktail) before intermittent sonication for 10 min at 4 °C. Chromatin was then cleared using protein A-sepharose (GE Healthcare) and equal amounts were incubated overnight at 4 °C on a rotator either with rabbit anti-TFEB antibody (Cell Signaling) or mouse anti-TFEB antibody (MyBioSource), anti-Histone H3 (Abcam) or anti-mouse- or anti-rabbit-unspecific IgGs (Sigma Aldrich). Immunocomplexes were subsequently isolated using protein A-sepharose (GE Healthcare) for 2 h at 4 °C, washed twice with buffer D (20 mM Tris pH 8.0, 2 mM EDTA, 50 mM NaCl, 1% Triton X-100, 0.1% SDS), once with buffer E (10 mM Tris pH 8.0, 1 mM EDTA, 0.25 M LiCl, 1% NP-40, 0.1% sodium deoxycholate monohydrate) and once with TE buffer. Samples were then eluted using buffer F (100 mM NaHCO_3_, 1% SDS). The crosslinking was reversed by treating the samples with RNase A and 0.3 M NaCl overnight at 67 °C. RNase A was then inactivated by incubating the samples with proteinase K (Fisher Scientific) for 2 h at 45 °C. Samples were then cleaned using the Qiaquick PCR purification kit (Qiagen) and used for real-time PCR analysis using the standard curve method. The primers used for the amplification of the putative TFEB-binding sites are listed in Supplementary Table 1.

### Statistical analysis

Significance levels for comparisons between groups were determined with unpaired or paired two-tailed Student's *t*-test using GraphPad Prism 5 (GraphPad Software) or Excel (Microsoft Office), where appropriate. Quantifications are plotted as mean±s.d. or s.e.m.

### Data availability

The authors declare that the data supporting the findings of this study are available within the article and its Supplementary Information files.

## Additional information

**How to cite this article:** Bento, C. F. *et al*. The Parkinson's disease-associated genes *ATP13A2* and *SYT11* regulate autophagy via a common pathway. *Nat. Commun.* 7:11803 doi: 10.1038/ncomms11803 (2016).

## Supplementary Material

Supplementary Information Supplementary Figures 1 - 7 and Supplementary Table 1

## References

[b1] Frake R. A., Ricketts T., Menzies F. M. & Rubinsztein D. C. Autophagy and neurodegeneration. J. Clin. Invest. 125, 65–74 (2015). 25654552 10.1172/JCI73944 PMC4382230

[b2] Schultz M. L., Tecedor L., Chang M. & Davidson B. L. Clarifying lysosomal storage diseases. Trends Neurosci. 34, 401–410 (2011). 21723623 10.1016/j.tins.2011.05.006 PMC3153126

[b3] Decressac M. . TFEB-mediated autophagy rescues midbrain dopamine neurons from alpha-synuclein toxicity. Proc. Natl Acad. Sci. USA 110, E1817–E1826 (2013). 23610405 10.1073/pnas.1305623110 PMC3651458

[b4] Dehay B. . Pathogenic lysosomal depletion in Parkinson's disease. J. Neurosci. 30, 12535–12544 (2010). 20844148 10.1523/JNEUROSCI.1920-10.2010 PMC6633458

[b5] Dehay B. . Loss of P-type ATPase ATP13A2/PARK9 function induces general lysosomal deficiency and leads to Parkinson disease neurodegeneration. Proc. Natl Acad. Sci. USA 109, 9611–9616 (2012). 22647602 10.1073/pnas.1112368109 PMC3386132

[b6] Park J. S. . Pathogenic effects of novel mutations in the P-type ATPase ATP13A2 (PARK9) causing Kufor-Rakeb syndrome, a form of early-onset parkinsonism. Hum. Mutat. 32, 956–964 (2011). 21542062 10.1002/humu.21527

[b7] Ramirez A. . Hereditary parkinsonism with dementia is caused by mutations in ATP13A2, encoding a lysosomal type 5 P-type ATPase. Nat. Genet. 38, 1184–1191 (2006). 16964263 10.1038/ng1884

[b8] Usenovic M., Tresse E., Mazzulli J. R., Taylor J. P. & Krainc D. Deficiency of ATP13A2 leads to lysosomal dysfunction, alpha-synuclein accumulation, and neurotoxicity. J. Neurosci. 32, 4240–4246 (2012). 22442086 10.1523/JNEUROSCI.5575-11.2012 PMC3462811

[b9] Gitler A. D. . Alpha-synuclein is part of a diverse and highly conserved interaction network that includes PARK9 and manganese toxicity. Nat. Genet. 41, 308–315 (2009). 19182805 10.1038/ng.300 PMC2683786

[b10] Lill C. M. . Comprehensive research synopsis and systematic meta-analyses in Parkinson's disease genetics: the PDGene database. PLoS Genet. 8, e1002548 (2012). 22438815 10.1371/journal.pgen.1002548 PMC3305333

[b11] Nalls M. A. . Imputation of sequence variants for identification of genetic risks for Parkinson's disease: a meta-analysis of genome-wide association studies. Lancet 377, 641–649 (2011). 21292315 10.1016/S0140-6736(10)62345-8 PMC3696507

[b12] Sharma M. . Large-scale replication and heterogeneity in Parkinson disease genetic loci. Neurology 79, 659–667 (2012). 22786590 10.1212/WNL.0b013e318264e353 PMC3414661

[b13] Nalls M. A. . Large-scale meta-analysis of genome-wide association data identifies six new risk loci for Parkinson's disease. Nat. Genet. 46, 989–993 (2014). 25064009 10.1038/ng.3043 PMC4146673

[b14] Inoue S. . Synaptotagmin XI as a candidate gene for susceptibility to schizophrenia. Am. J. Med. Genet. B Neuropsychiatr. Genet. 144B, 332–340 (2007). 17192956 10.1002/ajmg.b.30465

[b15] Usenovic M. . Identification of novel ATP13A2 interactors and their role in alpha-synuclein misfolding and toxicity. Hum. Mol. Genet. 21, 3785–3794 (2012). 22645275 10.1093/hmg/dds206 PMC3412378

[b16] Sudhof T. C. Calcium control of neurotransmitter release. Cold Spring Harb. Perspect. Biol. 4, a011353 (2012). 22068972 10.1101/cshperspect.a011353 PMC3249630

[b17] Arango Duque G., Fukuda M. & Descoteaux A. Synaptotagmin XI regulates phagocytosis and cytokine secretion in macrophages. J. Immunol. 190, 1737–1745 (2013). 23303671 10.4049/jimmunol.1202500

[b18] Sardiello M. . A gene network regulating lysosomal biogenesis and function. Science 325, 473–477 (2009). 19556463 10.1126/science.1174447

[b19] Palmieri M. . Characterization of the CLEAR network reveals an integrated control of cellular clearance pathways. Hum. Mol. Genet. 20, 3852–3866 (2011). 21752829 10.1093/hmg/ddr306

[b20] Settembre C. . A lysosome-to-nucleus signalling mechanism senses and regulates the lysosome via mTOR and TFEB. EMBO J. 31, 1095–1108 (2012). 22343943 10.1038/emboj.2012.32 PMC3298007

[b21] Martina J. A. & Puertollano R. Rag GTPases mediate amino acid-dependent recruitment of TFEB and MITF to lysosomes. J. Cell. Biol. 200, 475–491 (2013). 23401004 10.1083/jcb.201209135 PMC3575543

[b22] Thoreen C. C. . An ATP-competitive mammalian target of rapamycin inhibitor reveals rapamycin-resistant functions of mTORC1. J. Biol. Chem. 284, 8023–8032 (2009). 19150980 10.1074/jbc.M900301200 PMC2658096

[b23] Sancak Y. . Ragulator-Rag complex targets mTORC1 to the lysosomal surface and is necessary for its activation by amino acids. Cell 141, 290–303 (2010). 20381137 10.1016/j.cell.2010.02.024 PMC3024592

[b24] Klionsky D. J. . Guidelines for the use and interpretation of assays for monitoring autophagy. Autophagy 8, 445–544 (2012). 22966490 10.4161/auto.19496 PMC3404883

[b25] Han S. . Pam (protein associated with Myc) functions as an E3 ubiquitin ligase and regulates TSC/mTOR signaling. Cell Signal. 20, 1084–1091 (2008). 18308511 10.1016/j.cellsig.2008.01.020 PMC2435383

[b26] Inoki K., Li Y., Zhu T., Wu J. & Guan K. L. TSC2 is phosphorylated and inhibited by Akt and suppresses mTOR signalling. Nat. Cell Biol. 4, 648–657 (2002). 12172553 10.1038/ncb839

[b27] Kabeya Y. . LC3, a mammalian homologue of yeast Apg8p, is localized in autophagosome membranes after processing. EMBO J. 19, 5720–5728 (2000). 11060023 10.1093/emboj/19.21.5720 PMC305793

[b28] Kimura S., Noda T. & Yoshimori T. Dissection of the autophagosome maturation process by a novel reporter protein, tandem fluorescent-tagged LC3. Autophagy 3, 452–460 (2007). 17534139 10.4161/auto.4451

[b29] Ishidoh K. & Kominami E. Processing and activation of lysosomal proteinases. Biol. Chem. 383, 1827–1831 (2002). 12553719 10.1515/BC.2002.206

[b30] Turk V., Turk B. & Turk D. Lysosomal cysteine proteases: facts and opportunities. EMBO J. 20, 4629–4633 (2001). 11532926 10.1093/emboj/20.17.4629 PMC125585

[b31] Webb J. L., Ravikumar B., Atkins J., Skepper J. N. & Rubinsztein D. C. Alpha-Synuclein is degraded by both autophagy and the proteasome. J. Biol. Chem. 278, 25009–25013 (2003). 12719433 10.1074/jbc.M300227200

[b32] Settembre C. . TFEB links autophagy to lysosomal biogenesis. Science 332, 1429–1433 (2011). 21617040 10.1126/science.1204592 PMC3638014

[b33] Park J. S., Koentjoro B., Veivers D., Mackay-Sim A. & Sue C. M. Parkinson's disease-associated human ATP13A2 (PARK9) deficiency causes zinc dyshomeostasis and mitochondrial dysfunction. Hum. Mol. Genet. 23, 2802–2815 (2014). 24399444 10.1093/hmg/ddt623 PMC4014187

[b34] Tsunemi T. & Krainc D. Zn(2)(+) dyshomeostasis caused by loss of ATP13A2/PARK9 leads to lysosomal dysfunction and alpha-synuclein accumulation. Hum. Mol. Genet. 23, 2791–2801 (2014). 24334770 10.1093/hmg/ddt572 PMC4014186

[b35] Huynh D. P., Scoles D. R., Nguyen D. & Pulst S. M. The autosomal recessive juvenile Parkinson disease gene product, parkin, interacts with and ubiquitinates synaptotagmin XI. Hum. Mol. Genet. 12, 2587–2597 (2003). 12925569 10.1093/hmg/ddg269

[b36] Pawlyk A. C. . Novel monoclonal antibodies demonstrate biochemical variation of brain parkin with age. J. Biol. Chem. 278, 48120–48128 (2003). 12972409 10.1074/jbc.M306889200

[b37] Grenier K., McLelland G. L. & Fon E. A. Parkin- and PINK1-dependent mitophagy in neurons: will the real pathway please stand up? Front. Neurol. 4, 100 (2013). 23882257 10.3389/fneur.2013.00100 PMC3715719

[b38] Gusdon A. M., Zhu J., Van Houten B. & Chu C. T. ATP13A2 regulates mitochondrial bioenergetics through macroautophagy. Neurobiol. Dis. 45, 962–972 (2012). 22198378 10.1016/j.nbd.2011.12.015 PMC3291101

[b39] Pastore N. . Gene transfer of master autophagy regulator TFEB results in clearance of toxic protein and correction of hepatic disease in alpha-1-anti-trypsin deficiency. EMBO Mol. Med. 5, 397–412 (2013). 23381957 10.1002/emmm.201202046 PMC3598080

[b40] Spampanato C. . Transcription factor EB (TFEB) is a new therapeutic target for Pompe disease. EMBO Mol. Med. 5, 691–706 (2013). 23606558 10.1002/emmm.201202176 PMC3662313

[b41] Duvel K. . Activation of a metabolic gene regulatory network downstream of mTOR complex 1. Mol. Cell 39, 171–183 (2010). 20670887 10.1016/j.molcel.2010.06.022 PMC2946786

[b42] Hu G. . A conserved mechanism of TOR-dependent RCK-mediated mRNA degradation regulates autophagy. Nat. Cell Biol. 17, 930–942 (2015). 26098573 10.1038/ncb3189 PMC4528364

[b43] Klionsky D. J., Elazar Z., Seglen P. O. & Rubinsztein D. C. Does bafilomycin A1 block the fusion of autophagosomes with lysosomes? Autophagy 4, 849–850 (2008). 18758232 10.4161/auto.6845

[b44] Bar-Peled L. & Sabatini D. M. Regulation of mTORC1 by amino acids. Trends Cell Biol. 24, 400–406 (2014). 24698685 10.1016/j.tcb.2014.03.003 PMC4074565

[b45] Hu Y. . Lysosomal pH plays a key role in regulation of mTOR activity in osteoclasts. J. Cell Biochem. 117, 413–425 (2016). 26212375 10.1002/jcb.25287

[b46] Newton P. T., Vuppalapati K. K., Bouderlique T. & Chagin A. S. Pharmacological inhibition of lysosomes activates the MTORC1 signaling pathway in chondrocytes in an autophagy-independent manner. Autophagy 11, 1594–1607 (2015). 26259639 10.1080/15548627.2015.1068489 PMC4590675

[b47] Zoncu R. . mTORC1 senses lysosomal amino acids through an inside-out mechanism that requires the vacuolar H(+)-ATPase. Science 334, 678–683 (2011). 22053050 10.1126/science.1207056 PMC3211112

[b48] Sudhof T. C. Synaptotagmins: why so many? J. Biol. Chem. 277, 7629–7632 (2002). 11739399 10.1074/jbc.R100052200

[b49] von Poser C., Ichtchenko K., Shao X., Rizo J. & Sudhof T. C. The evolutionary pressure to inactivate. A subclass of synaptotagmins with an amino acid substitution that abolishes Ca2+ binding. J. Biol. Chem. 272, 14314–14319 (1997). 9162066 10.1074/jbc.272.22.14314

[b50] Dai H. . Structural basis for the evolutionary inactivation of Ca2+ binding to synaptotagmin 4. Nat. Struct. Mol. Biol. 11, 844–849 (2004). 15311271 10.1038/nsmb817

[b51] Wang C. . Synaptotagmin-11 inhibits clathrin-mediated and bulk endocytosis. EMBO Rep. 17, 47–63 (2016). 26589353 10.15252/embr.201540689 PMC4718405

[b52] Matheoud D. . Leishmania evades host immunity by inhibiting antigen cross-presentation through direct cleavage of the SNARE VAMP8. Cell Host Microbe 14, 15–25 (2013). 23870310 10.1016/j.chom.2013.06.003

[b53] Vinet A. F., Fukuda M., Turco S. J. & Descoteaux A. The Leishmania donovani lipophosphoglycan excludes the vesicular proton-ATPase from phagosomes by impairing the recruitment of synaptotagmin V. PLoS Pathog. 5, e1000628 (2009). 19834555 10.1371/journal.ppat.1000628 PMC2757729

[b54] Larsen K. B. . A reporter cell system to monitor autophagy based on p62/SQSTM1. Autophagy 6, 784–793 (2010). 20574168 10.4161/auto.6.6.12510

[b55] Manning B. D., Tee A. R., Logsdon M. N., Blenis J. & Cantley L. C. Identification of the tuberous sclerosis complex-2 tumor suppressor gene product tuberin as a target of the phosphoinositide 3-kinase/akt pathway. Mol. Cell 10, 151–162 (2002). 12150915 10.1016/s1097-2765(02)00568-3

[b56] Jimenez-Sanchez M. . siRNA screen identifies QPCT as a druggable target for Huntington's disease. Nat. Chem. Biol. 11, 347–354 (2015). 25848931 10.1038/nchembio.1790 PMC4696152

[b57] Ran F. A. . Double nicking by RNA-guided CRISPR Cas9 for enhanced genome editing specificity. Cell 154, 1380–1389 (2013). 23992846 10.1016/j.cell.2013.08.021 PMC3856256

[b58] Ran F. A. . Genome engineering using the CRISPR-Cas9 system. Nat. Protoc. 8, 2281–2308 (2013). 24157548 10.1038/nprot.2013.143 PMC3969860

[b59] Cost G. J. & Cozzarelli N. R. Directed assembly of DNA molecules via simultaneous ligation and digestion. BioTechniques 42, (84): 86–89 (2007). 10.2144/000112283 17269489

[b60] Renna M. . Autophagic substrate clearance requires activity of the syntaxin-5 SNARE complex. J. Cell. Sci. 124, 469–482 (2011). 21242315 10.1242/jcs.076489 PMC3022001

