# Supplementary material for: The Parkinson's disease-associated genes ATP13A2 and SYT11 regulate autophagy via a common pathway
Source: Nat Commun. 2016 Jun 9;7:11803. doi: 10.1038/ncomms11803 (PMC4906231; doi:10.1038/ncomms11803)
Supplement: Supplementary Information — Supplementary Figures 1 - 7 and Supplementary Table 1 [file ncomms11803-s1.pdf]

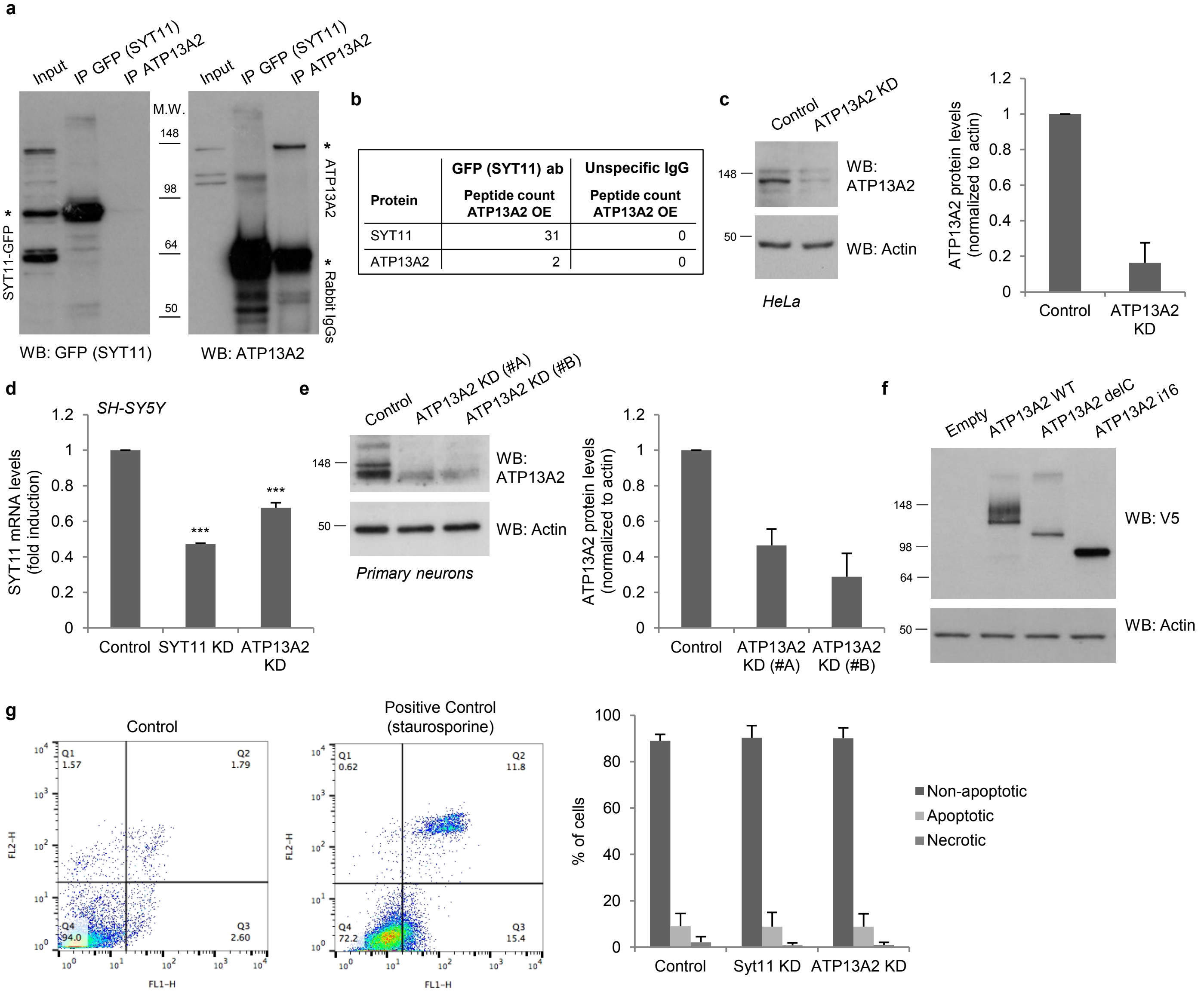

Protein	GFP (SYT11) ab<br>Peptide count ATP13A2 OE	Unspecific IgG<br>Peptide count ATP13A2 OE
SYT11	31	0
ATP13A2	2	0

Supplementary figure 1. (a and b) Our immunoprecipitation attempts were not able to detect a physical interaction between SYT11 and ATP13A2 by western blotting and only retrieved two ATP13A2 peptides by mass spectrometry analysis of SYT11-co-immunoprecipitated proteins. GFP-tagged SYT11 overexpressing HeLa cells were immunoprecipitated using antibodies against GFP or ATP13A2 (a). Immunoprecipitates and respective whole cell lysates were blotted against GFP and ATP13A2. (b)Table summarizing peptide counts of ATP13A2 that co-immunoprecipitate with GFP-SYT11. Lysates of HeLa cells transfected with GFP-tagged SYT11 and V5-tagged ATP13A2 were used to GFP-immunoprecipitation followed by mass spectrometry analysis of co-immunoprecipitated proteins. **(c)** Western blot against ATP13A2 and actin confirming the knockdown efficiency of the siRNAs against ATP13A2 after 96 hours of transfection. The quantification represents the mean ± S.D. of three independent experiments. **(d)** Human neuroblastoma SH-SY5Y cells transfected with Control, SYT11 siRNA or ATP13A2 siRNA were used to measure the mRNA levels of SYT11 and GAPDH by real-time PCR. The graph represents the mean ± S.D. of one experiment with triplicates each condition. *** $p < 0.001$ (two-tailed unpaired Student's *t*-test). **(e)** Western blot against ATP13A2 and actin confirming the knockdown efficiency of the lentiviral shRNAs against ATP13A2 in mouse primary neuron cultures after 5 days of transduction. The quantification represents the mean ± S.D. of three independent experiments. **(f)** Western blot against V5 and actin confirming the overexpression efficiencies of V5-tagged ATP13A2 WT, ATP13A2 delC or ATP13A2 i16 constructs after 24 hours of transfection. **(g)** HeLa cells transfected with Control, SYT11 siRNA or ATP13A2 siRNA were used to determine cellular apoptosis by flow cytometry using the AnnexinV-FITC/PI assay. Staurosporine (5 µg/ml for 4 hours) was used as a positive control.

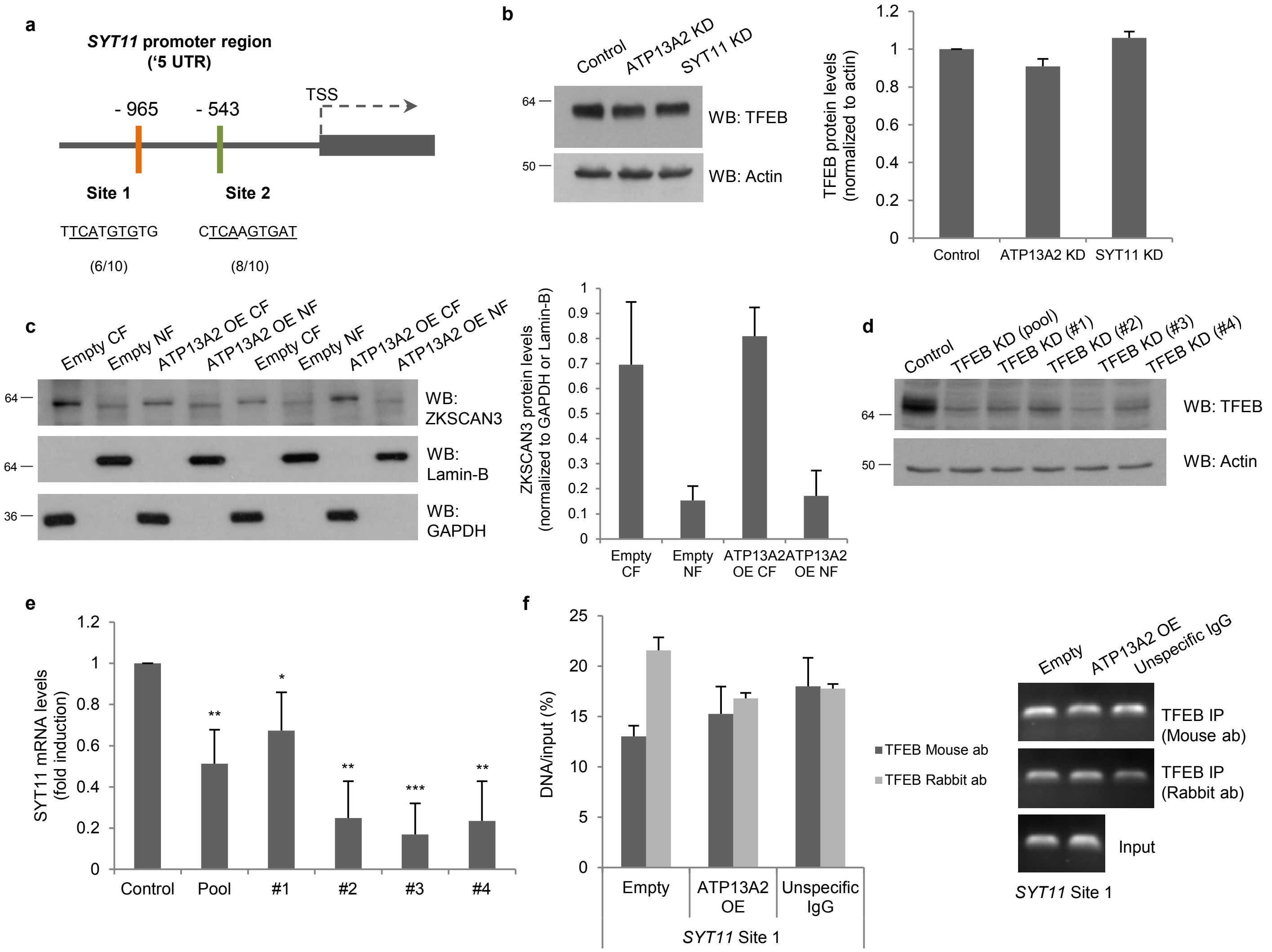

Supplementary figure 2. **(a)** Diagram showing the position and the nucleotide sequence of the putative TFEB-binding motifs in the human *SYT11* promoter. 6 in 10 and 8 in 10 nucleotides are shared by Site1 and Site2 of *SYT11* promoter with the CLEAR consensus sequence 5'-GTCACGTGAC-3', respectively. **(b)** Lysates of HeLa cells transfected with Control, SYT11 siRNA or ATP13A2 siRNA were blotted against TFEB and actin. The quantification represents the mean ± S.D. of three experiments. **(c)** Control and ATP13A2-overexpressing HeLa cells were used for cytosolic and nuclear fractionation. The different fractions were blotted for ZKSCAN3, Lamin-B and GAPDH. The quantification represents the mean ± S.D. of three experiments. **(d)** Western blot against TFEB and actin confirming the efficiency of the used siRNAs in knocking-down ATP13A2 after 96 hours of transfection. **(e)** HeLa cells transfected with Control or TFEB siRNA (pool or deconvoluted oligos) were used to measure the mRNA levels of SYT11 and GAPDH by real-time PCR. The graph represents the mean ± S.D. of one experiment with triplicates each condition. A representative experiment of 2 independent experiments similarly significant is shown. * $p < 0.05$, ** $p < 0.01$, *** $p < 0.001$ (two-tailed unpaired Student's *t*-test). **(f)** Control and ATP13A2 WT-overexpressing HeLa cells were used to perform chromatin immunoprecipiation (ChIP). Two different antibodies were tested for TFEB immunoprecipitation. Real-time PCR was performed using primers for the putative TFEB-binding Site 1 on the promoter of *SYT11*. The graph represents the mean ± S.D. of one experiment with triplicates. A representative experiment of 2 independent experiments is shown (left). Amplification products of the qPCR were run in a 1% agarose gel (right). Due to high IgG unspecific binding to the chromatin region of Site 1, we could not confirm Site 1 as a TFEB-binding site in *SYT11* promoter.

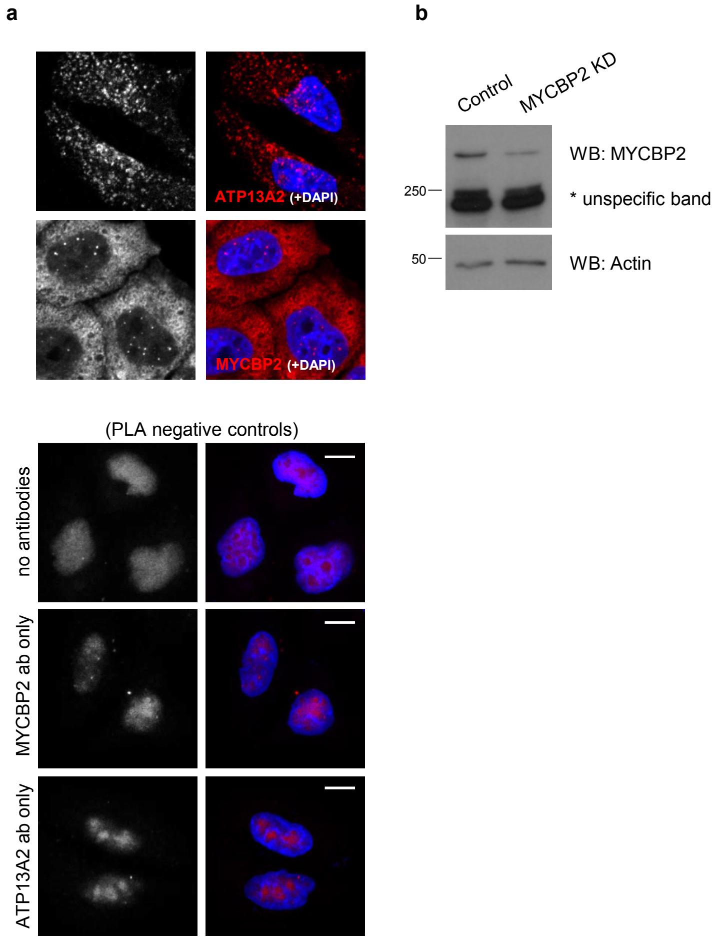

Supplementary figure 3. (a) HeLa cells were fixed and analysed by immunofluorescence using primary antibodies against ATP13A2 and MYCBP2 (top panel). These antibodies were used for proximity ligation assays shown in the main figures. Negative controls of the proximity ligation assays for ATP13A2 and MYCBP2 were performed where antibodies were individually tested or no antibody was added (bottom panel) (scale bar 10 μM). **(b)** Western blot against MYCBP2 and actin confirming the MYCBP2 knockdown efficiency produced by the siRNAs used in experiments shown in the main figures.

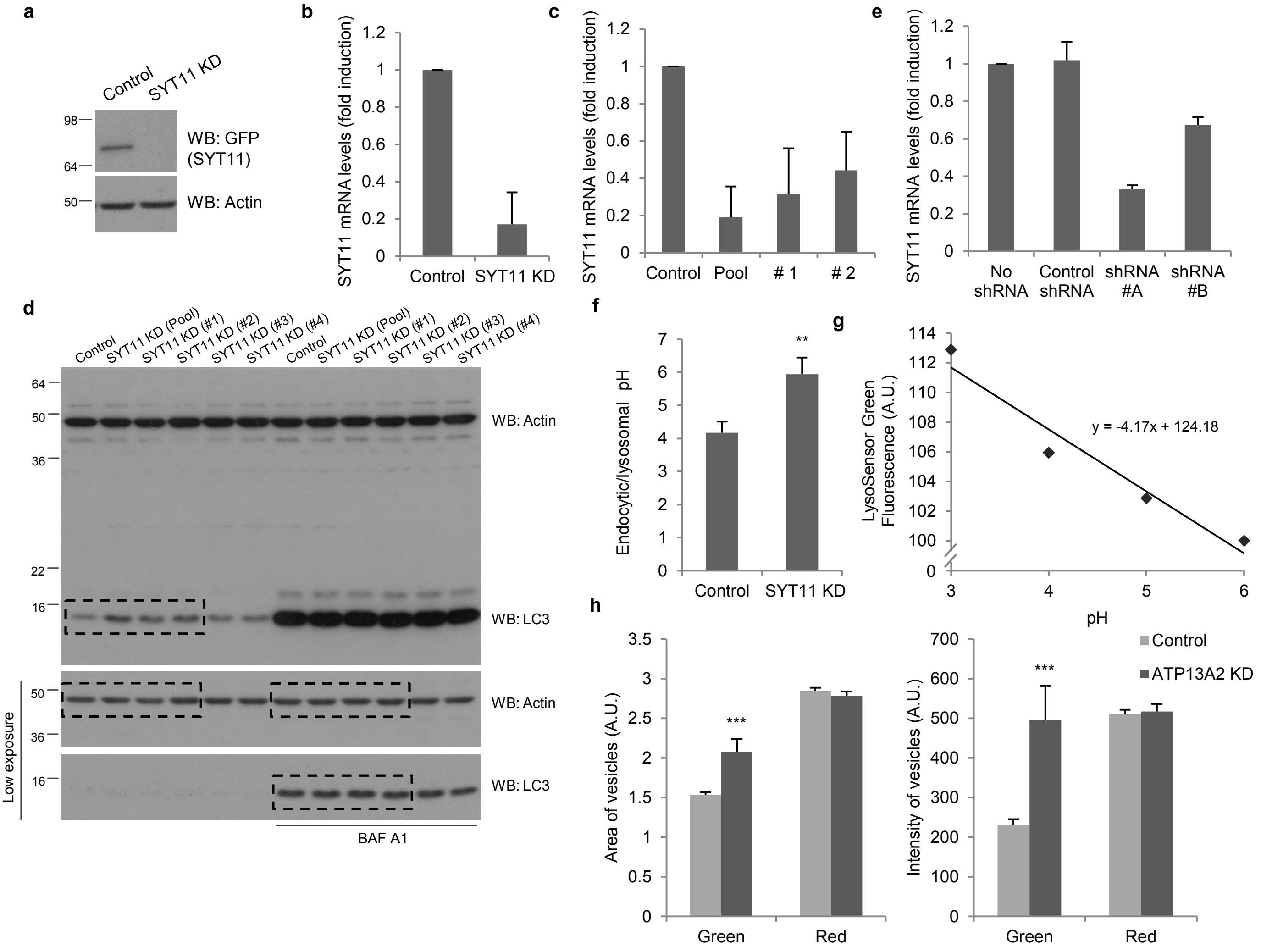

Supplementary figure 4. (a) Western blot against GFP and actin confirming the knockdown efficiency of our siRNAs against transfected GFP-SYT11 after 96 hours of siRNA transfection. **(b and c)** HeLa cells transfected with Control or SYT11 siRNA (pool or deconvoluted oligos) were used to measure the mRNA levels of SYT11 and GAPDH by real-time PCR. The graphs show the mean ± S.D. of three independent experiments. **(d)** Full blot image of Fig. 5b - although samples with and without BAF A1 were run in the same gel, in order to test the efficiency of BAF A1 treatment to increase the levels of LC3-II, we show distinct panels in the main figure to avoid overexposure of the chemiluminescent signal. **(e)** Primary cultures of neurons were transduced with Control or SYT11 lentiviral shRNAs for 5 days. In order to test the knockdown efficiency of each shRNA, the mRNA levels of SYT11 and GAPDH were measured by real-time PCR. The graph shows the mean ± S.D. of one experiment with triplicates. **(f and g)** Control and SYT11-knockdown HeLa cells (~ 95% confluence) were incubated with 1 µM LysoSensor Green DND-189 for 30 minutes at 37°C. The endo-lysosomal pH was determined using a calibration curve (shown in g). The graph represents the mean ± S.D. of 3 independent experiments with 6 replicates for each condition. **(h)** Fixed HeLa cells stably expressing tandem fluorescent-tagged LC3 (mRFP-EGFP-LC3) transfected with Control or ATP13A2 siRNA were analysed on an automated ArrayScan system. Averages of vesicle size and intensity are shown in the graphs. Approximately 2000 cells were analysed per condition. The graph shows mean ± S.E.M. An increase of area and intensity of green vesicles suggests blockage of autophagosome degradation.

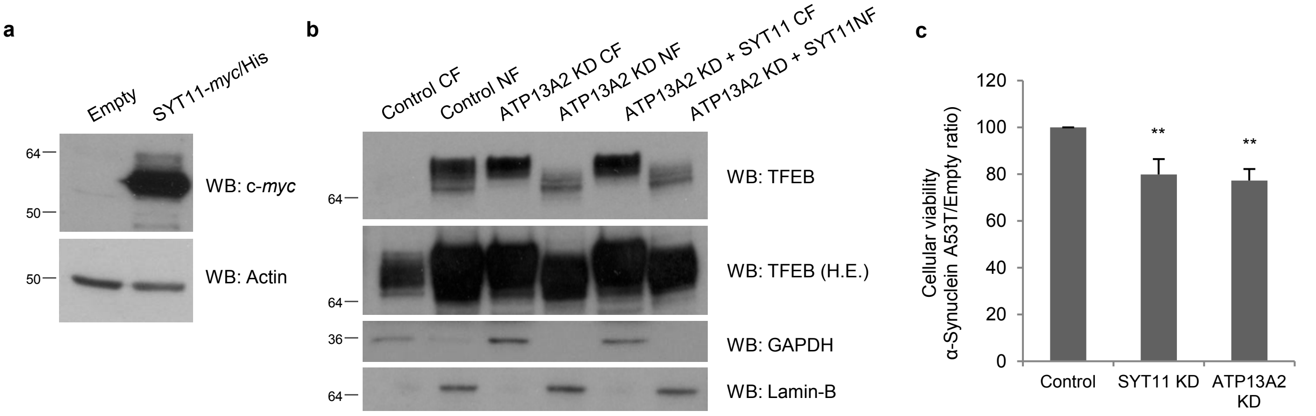

Supplementary figure 5. (a) HeLa cells were transfected with empty pcDNA3.1-*myc*/His or pcDNA3.1-SYT11-*myc*/His and lysates were blotted against c-*myc* and actin. **(b)** Control and ATP13A2-knockdown HeLa cells transfected with empty pEGFP or pEGFP-SYT11 in the last 30 hours of the experiment were used for cytosolic and nuclear fractionation. The different fractions were blotted for TFEB, Lamin-B and GAPDH; H.E. high exposure. **(c)** HeLa cells transfected with Control, SYT11 siRNA or ATP13A2 siRNA and empty pEGFP or GFP-α-synuclein A53T were used to measure cellular viability. The graph shows the mean ± S.D. of one experiment with 12 replicates and results were normalized to empty pEGFP condition. A representative experiment of 2 independent experiments is shown. ** $p < 0.01$ (two-tailed paired Student's *t*-test).

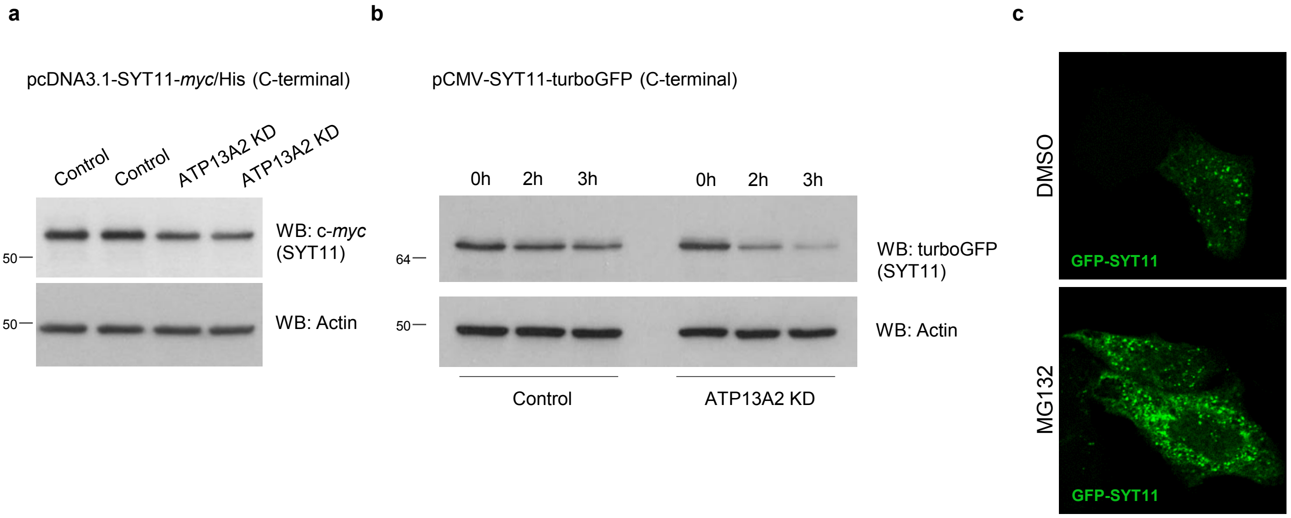

Supplementary figure 6. (**a)** Lysates of control and ATP13A2-knockdown HeLa cells transfected with empty pcDNA3.1-*myc*/His or pcDNA3.1-SYT11-*myc*/His were blotted against c-*myc* and actin. **(b)** Control and ATP13A2-knockdown HeLa cells were transfected with pCMV-SYT11-turboGFP for the last 24 hours. In the last 3 hours of the experiment, cells were treated with 50 µg/ml cycloheximide for the indicated time points. Cell lysates were blotted against turboGFP and actin. **(c)** HeLa cells transfected with pEGFP-SYT11 were treated with 10 µM MG132 (or DMSO) and imaged by fluorescence microscopy.

Supplementary figure 7.

Fig. 1c

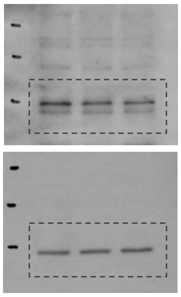

SYT11

Actin

Fig. 2a

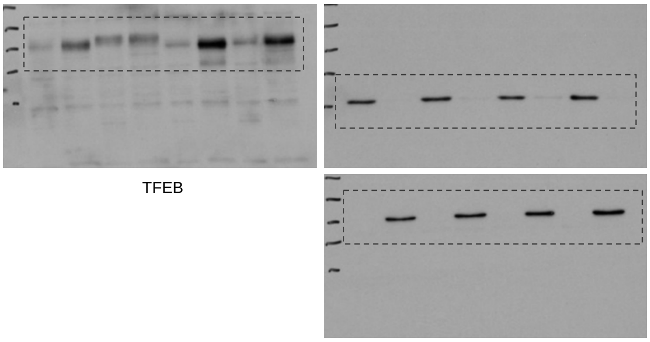

TFEB

GAPDH

Lamin-B

Fig. 2b

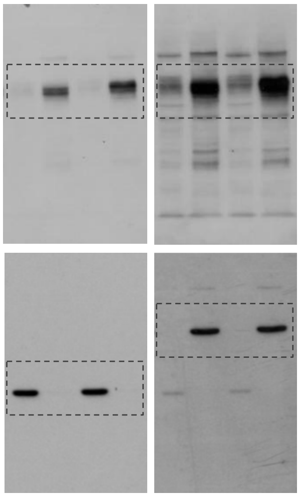

TFEB

GAPDH

Lamin-B

Fig. 2d

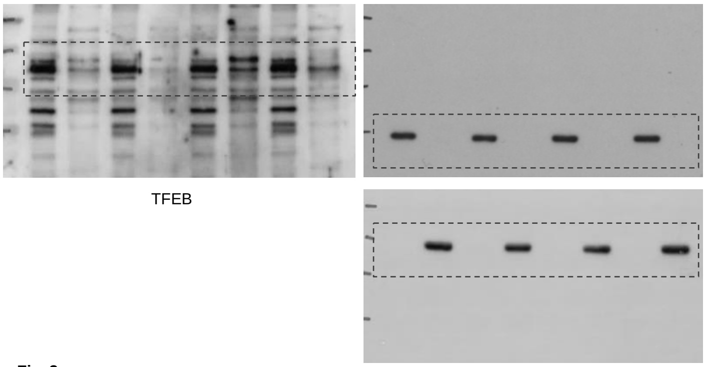

TFEB

GAPDH

Lamin-B

Fig. 3b

KD

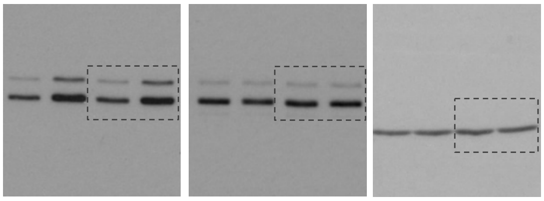

Phospho-p70S6K p70S6K Actin

OE

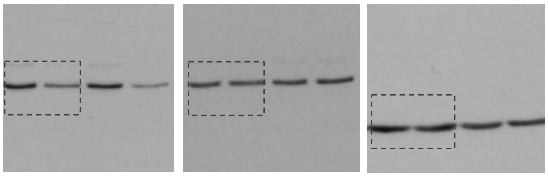

Phospho-p70S6K p70S6K Actin

Fig. 3c

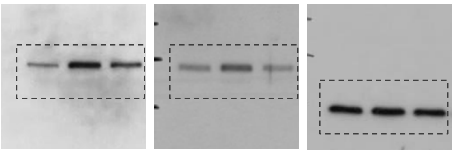

Phospho-p70S6K p70S6K Actin

Fig. 3d

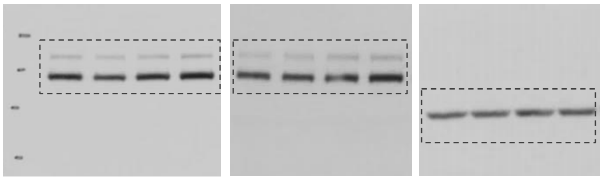

Phospho-p70S6K p70S6K Actin

Fig. 4b

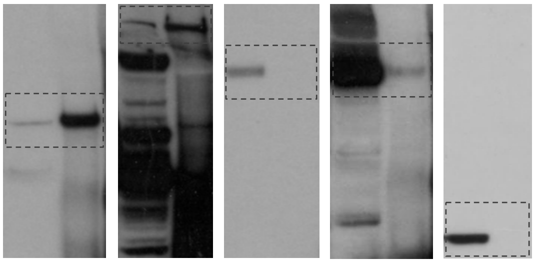

ATP13A2 MYCBP2 TSC2 TSC2 (high exposure) Actin

Fig. 4c

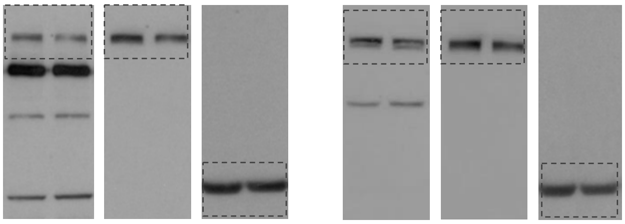

phospho Ser1387 TSC2 TSC2 Actin phospho Thr1462 TSC2 TSC2 Actin

Fig. 4d

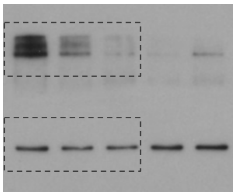

TSC2

Actin

Supplementary figure 7. continued

Fig. 4e

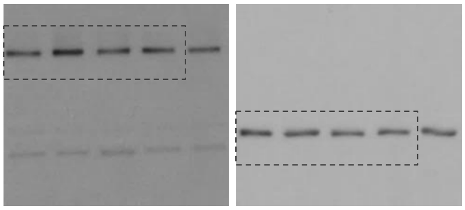

TSC2 Actin

Fig. 4f

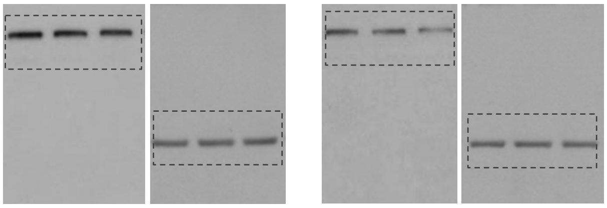

TSC2 Actin TSC2 Actin

Fig. 4g

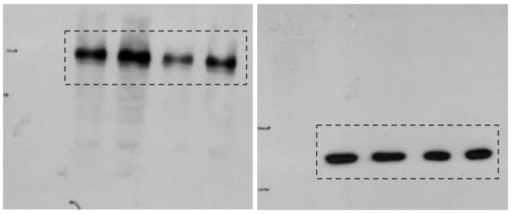

TSC2 Actin

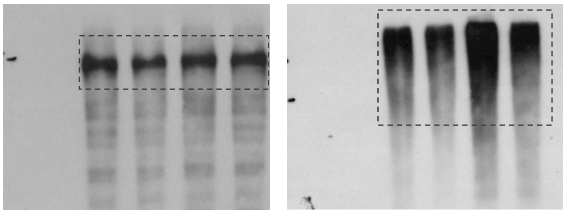

TSC2 HA (Ubiquitin)

Fig. 4i

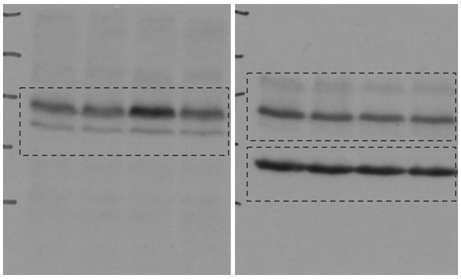

p70S6K

Actin

Phospho-p70S6K

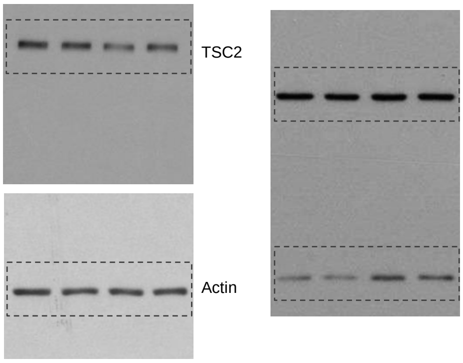

TSC2

Actin

Actin

LC3-II

Fig. 4h

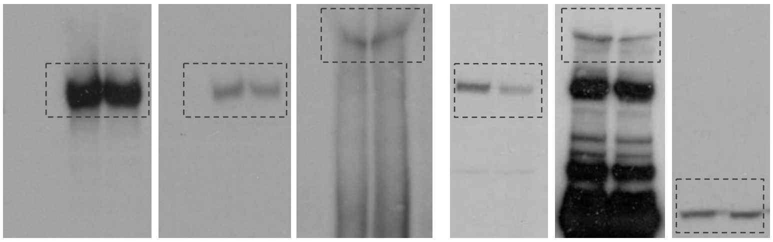

TSC2 (high exposure) TSC2 MYCBP2 TSC2 MYCBP2 Actin

Fig. 5c

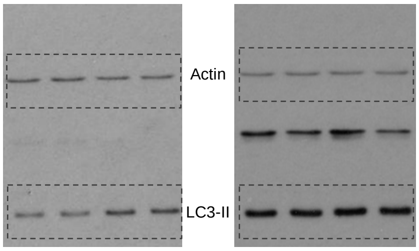

Actin

LC3-II

Fig. 5d

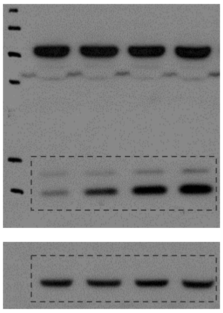

Actin

LC3

Actin

Fig. 5e

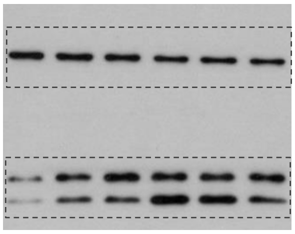

Actin

LC3

Fig. 5f

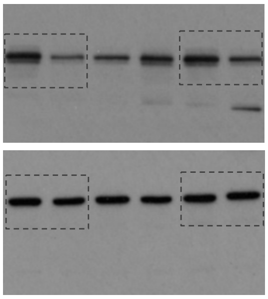

GFP (P62)

Actin

Fig. 5e

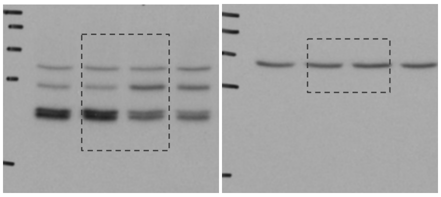

Cathepsin-L Actin

Fig. 6f

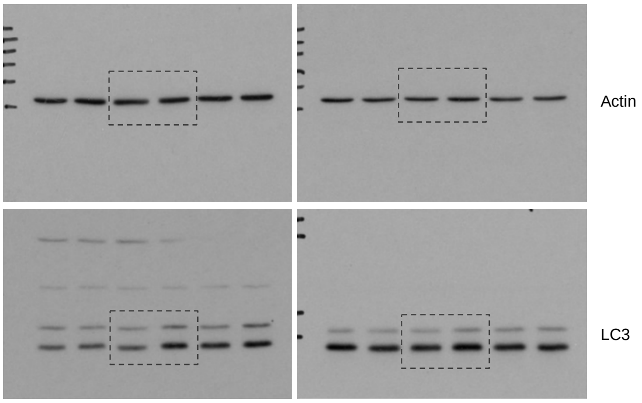

Fig. 6g

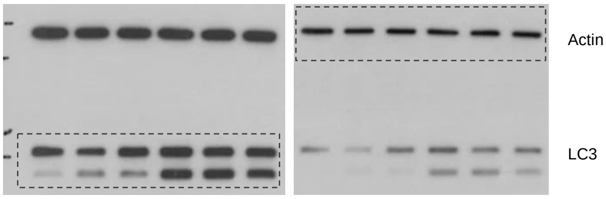

Fig. 7a

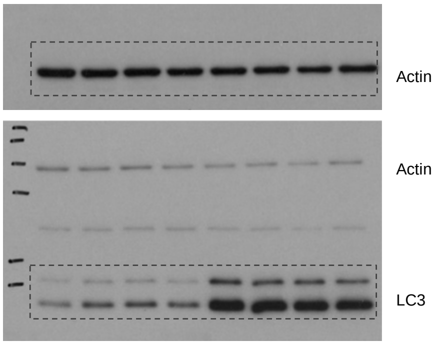

Fig. 7b

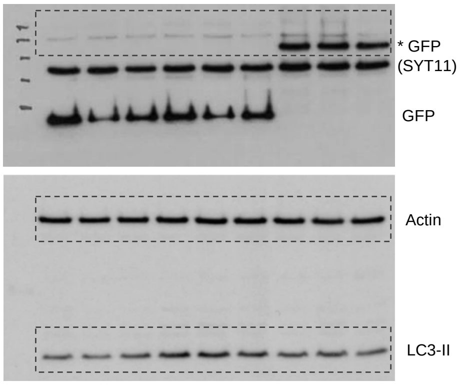

Fig. 7c

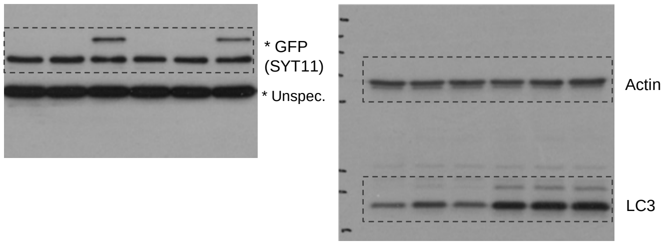

Fig. 7d

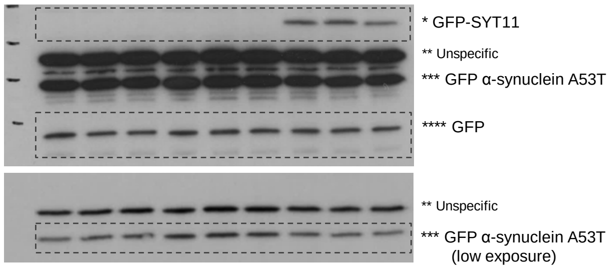

Fig. 7f

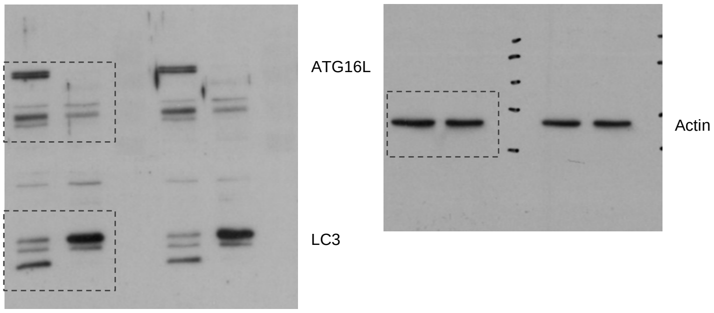

Fig. 7e

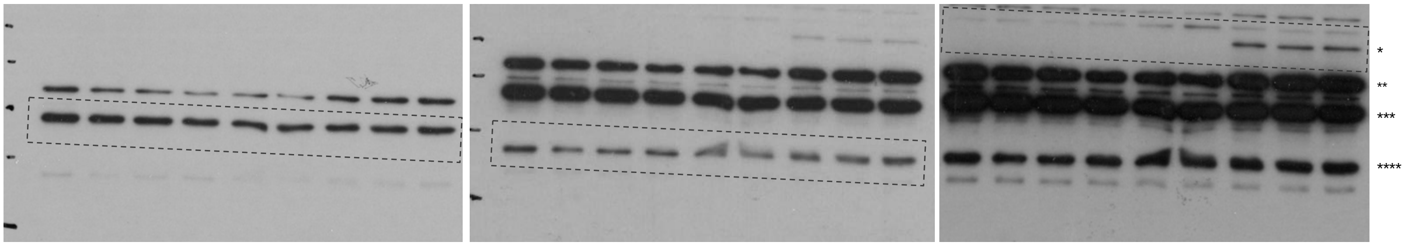

* GFP-SYT11

** Unspecific

*** GFP α-synuclein A53T

**** GFP

Supplementary figure 7. continued

Fig. 7h

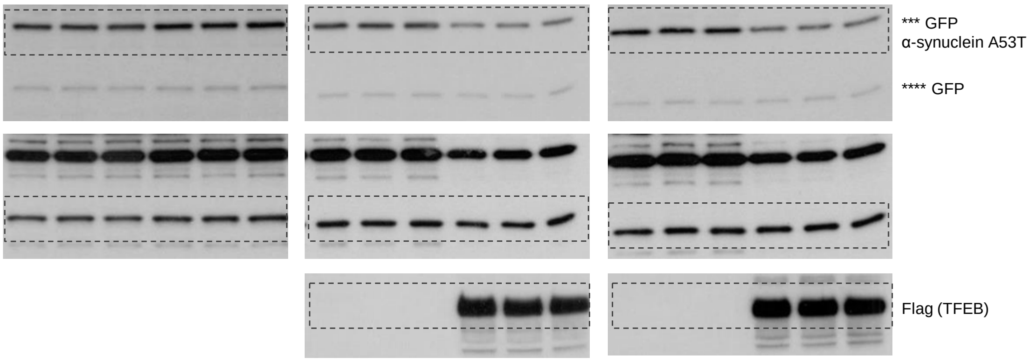

Fig. 8a

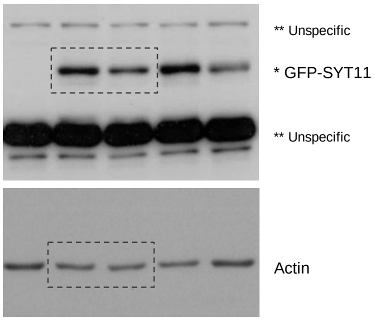

Fig. 8b

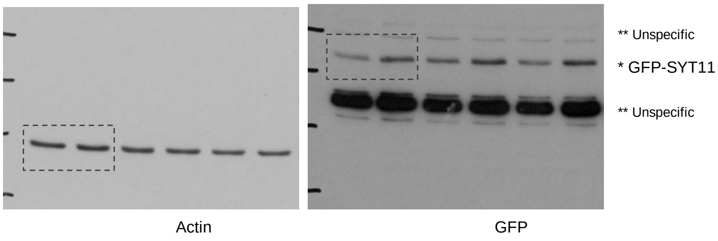

Fig. 8c

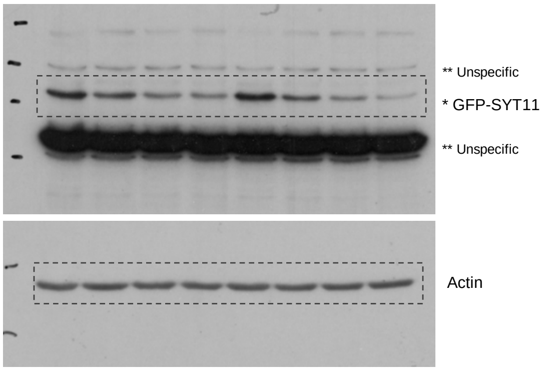

Fig. 8d

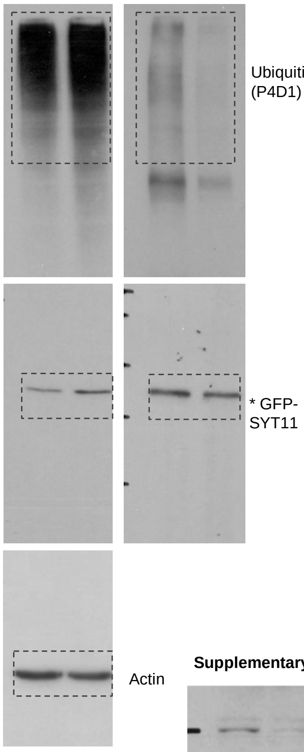

Fig. 8e

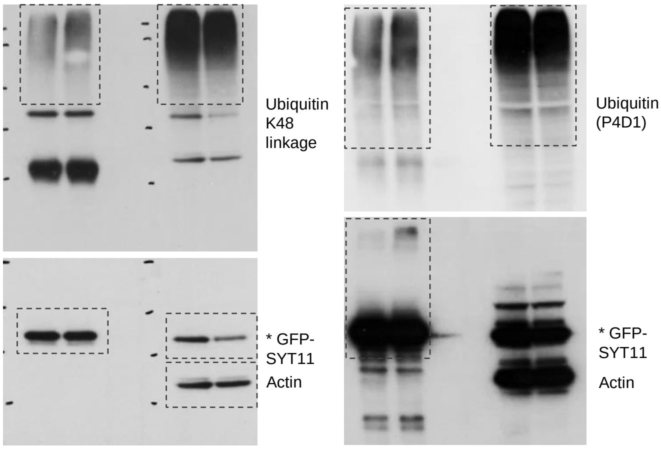

Fig. 8f

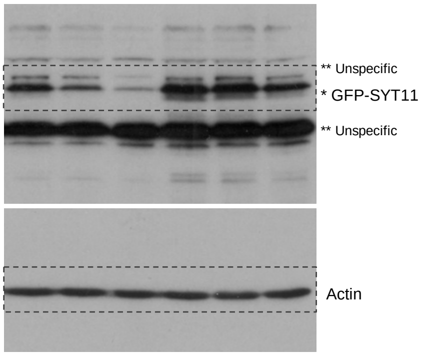

Supplementary fig. 1c

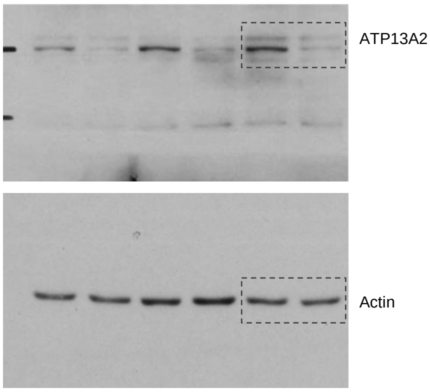

Supplementary fig. 1e

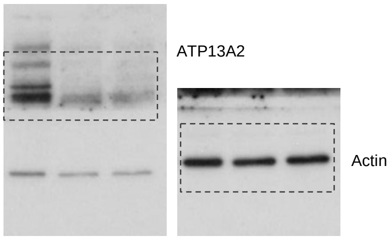

Supplementary fig. 1f

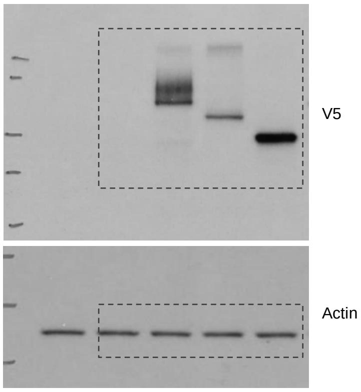

Supplementary figure 7. continued

Supplementary fig. 2b

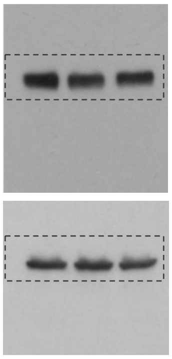

Supplementary fig. 2c

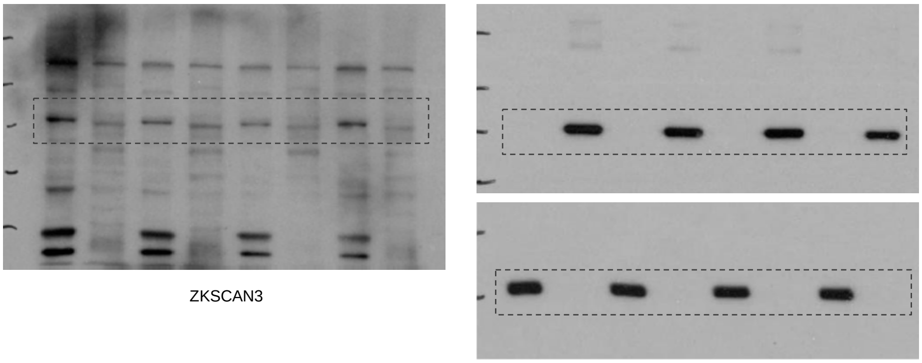

Supplementary fig. 2d

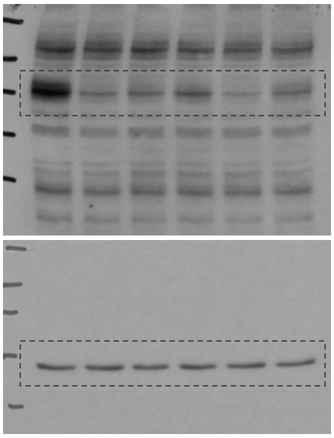

Supplementary fig. 3b

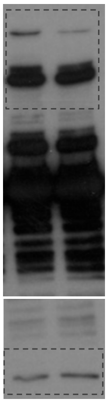

Supplementary fig. 4a

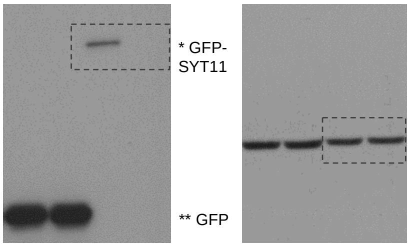

Supplementary fig. 5a

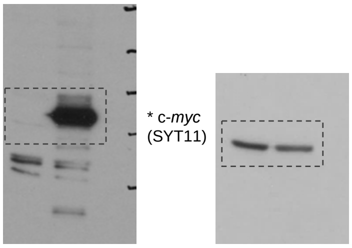

Supplementary fig. 5b

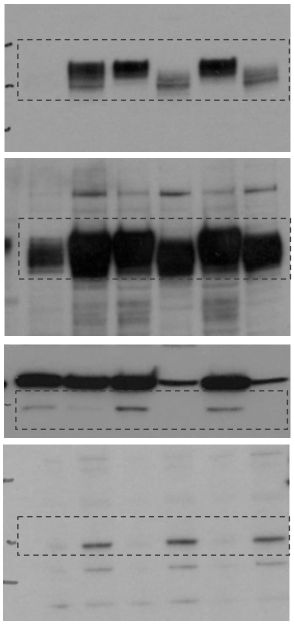

Supplementary fig. 6a

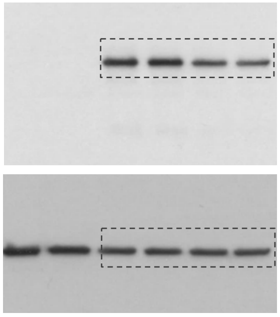

Supplementary fig. 6b

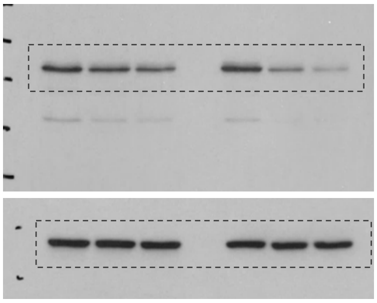

Supplementary table 1. Primers used for mutagenesis, real-time PCR and chromatin immunoprecipitation (ChIP)

Method	Gene	Set of primers
**Mutagenesis**	**human*SYT11*** (CLEAR Site-2)	FW 5'-CTCGAACTCCTGACCAAAAGAAATCTGCCCGCCTTGGCC-3'<br>RV 5'-GGCCAAGGCGGGCAGATTTCTTTTGGTCAGGAGTTCGAG-3'

Method	Gene	Set of primers
**Real-time PCR**	**human *SYT11***	FW 5'-CGCTTCTCTCGGGATGATGT-3'<br>RV 5'-CTCTGCTGATGCACTTCTGGAT-3'
	**human *GAPDH***	FW 5'-ACAGTCAGCCGCATCTTCTTT-3'<br>RV 5'-CAATACGACCAAATCCGTTGACT-3'
	**mouse *SYT11***	FW 5' - ATACGCCCCAGCTTTGATGT - 3'<br>RV 5' - CTTGTATGGCGGGGTCTTGT - 3'
	**mouse *GAPDH***	FW 5'-AAGGGCTCATGACCACAGTC-3'<br>RV 5'-ATCACGCCACAGCTTTCCA-3'

Method	Gene	Set of primers	Sequence position (related to TSS)
**ChIP**	**human *SYT11***	FW 5'-TATGCAAACATGGCAGTAGTC-3'<br>RV 5'-TGCTTAGGGTTTCGTATGAG-3'	Site 1 -965bp (the amplicon spans the putative TFEB-binding domain)
	**human *SYT11***	FW 5'-TAAGACAGTTTCCATCCACTG-3'<br>RV 5'-CTGATTTCAGTTTTCTGGGC-3'	Site 2 -543bp (#A) (the amplicon is adjacent to the TFEB-binding domain)
	**human *SYT11***	FW 5'-AACAGCTTTGCATACCACAATATCC-3'<br>RV 5'-CAGTGGATGGAAACTGTCTTAAACC-3'	Site 2 -543bp (#B) (the amplicon spans the TFEB-binding domain)
